# Elevated α5 integrin expression on myeloid cells in motor areas in amyotrophic lateral sclerosis is a therapeutic target

**DOI:** 10.1073/pnas.2306731120

**Published:** 2023-07-31

**Authors:** Aude Chiot, Shanu F. Roemer, Lisa Ryner, Alina Bogachuk, Katie Emberley, Dillon Brownell, Gisselle A. Jimenez, Michael Leviten, Randall Woltjer, Dennis W. Dickson, Lawrence Steinman, Bahareh Ajami

**Affiliations:** ^a^Department of Molecular Microbiology and Immunology, Oregon Health and Science University, Portland, OR 97239; ^b^Department of Behavioral and Systems Neuroscience, Oregon Health and Science University, Portland, OR 97239; ^c^Department of Neuroscience, Mayo Clinic, Jacksonville, FL 32224; ^d^Pasithea Therapeutics, Molecular Research Laboratories, South San Francisco, CA 94080; ^e^Jungers Center for Neurosciences Research, Department of Neurology, Oregon Health and Science University, Portland, OR 97239; ^f^Department of Pathology, Oregon Health and Science University, Portland, OR 97239; ^g^Department of Neurology and Neurological Sciences, Stanford University School of Medicine, Stanford, CA 94305

**Keywords:** amyotrophic lateral sclerosis, macrophages, microglial cells, α5 integrintherapeutic

## Abstract

Amyotrophic lateral sclerosis (ALS) is a fatal disease affecting upper and lower motor neurons. Microglia directly interact with motor neurons and participate in the progression of ALS. Single-cell mass cytometry (CyTOF) analysis revealed prominent expression of α5 integrin in microglia and macrophages in a superoxide dismutase-1 G93A mouse model of ALS (SOD1^G93A^). In postmortem tissues from ALS patients with various clinical ALS phenotypes and disease duration, α5 integrin is prominent in motor pathways of the central and peripheral nervous system and in perivascular zones associated with the blood–brain barrier. In SOD1^G93A^ mice, administration of a monoclonal antibody against α5 integrin increased survival compared to an isotype control and improved motor function on behavioral testing. Together, these findings in mice and in humans suggest that α5 integrin is a potential therapeutic target in ALS.

## Significance

Amyotrophic lateral sclerosis (ALS) is a fatal disease leading to progressive paralysis with no cure to date. Microglial cells and peripheral macrophages are known to be involved in disease progression and motor neuron degeneration. By assessing ALS mouse and ALS postmortem tissues, we demonstrate that α5 integrin, expressed by microglial cells and macrophages, is highly up-regulated in the spinal cord and peripheral nerves during ALS. Here, we propose to target α5 integrin as an immunomodulatory treatment for ALS. We show that the administration of a monoclonal antibody against α5 integrin to SOD1^G93A^ mice protects motor functions, delays disease progression, and increases mouse survival. Our work demonstrates the potential of targeting integrins in the neurodegenerative context.

Amyotrophic lateral sclerosis (ALS) is a neurodegenerative disease characterized by progressive motor neuron loss in the motor cortex, brainstem, and spinal cord, leading to fatal paralysis. Currently, there are three disease-modifying treatments approved for ALS in the United States (1). Although motor neuron degeneration is the hallmark of this disease, several studies indicate that the neurodegeneration in both familial and sporadic ALS is influenced by other cell types, including immune cells from the blood and glial cells from the central nervous system (CNS) and from the peripheral nervous system, challenging the neuron-centric theory in the pathogenesis of ALS (2–8).

Evidence from animal studies and from ALS patients has demonstrated that the degeneration and death of motor neurons is accompanied by inflammation (9–13). Genetic forms of ALS, which represent 10 to 20% of ALS cases, have been linked to several genes associated with inflammation, including *C9ORF72*, *TBK1,* and *OPTN* (14). Mice lacking C9orf72, Tbk1, or Optn showed changes of myeloid cell inflammatory profiles, including a higher propensity to produce proinflammatory cytokines and broad dysregulation of the immune system (15–19).

Whether inflammation has a correlative or causative role in the pathogenesis of ALS has been debated. Our previous study using a parabiosis model in mutant SOD1 mice (mSOD1), an animal model of ALS, demonstrated that inflammation in the spinal cord was primarily mediated by resident microglial cells as opposed to peripherally derived myeloid cells (20). A causative role for neuroinflammation is suggested by several studies. Selective deletion of SOD1 in CD11b^+^ cells, which includes microglia and peripheral myeloid cells, resulted in improved survival of mSOD1 mice (4). Similarly, when the microglial compartment of mutant SOD1 mice was reconstituted with wild-type microglia, disease progression was slowed, and survival was extended (3). Furthermore, depletion of myeloid cells, including microglia, by administration of a tyrosine kinase inhibitor (GW2580) attenuated motor neuron cell death, slowed disease progression, and extended life expectancy in mSOD1 mice (21). Recently, we have shown that replacement of endogenous mutated peripheral macrophages with neurotrophic macrophages increased survival of mSOD1 mice (8). These studies demonstrate that modulation of inflammation, both peripherally and centrally, represents a potential therapeutic approach for ALS.

In the present study, a systems immunology analysis utilizing mass cytometry (CyTOF) identified α5 integrin in microglia and peripheral nerve macrophages with distinct proinflammatory properties in the late stage of SOD1^G93A^ mice. The distribution of α5 integrin was studied in human autopsy material from ALS patients (sporadic and genetic forms of ALS, including *C9ORF72*, *SOD1*, *TARDBP,* and *TBK1*) and in SOD1^G93A^ mice. There was a striking spatial zonation of α5 integrin expression that was confined to the primary motor cortex and spinal cord, but it was sparse in sensory pathways and sensory neurons, providing evidence that α5 integrin is predominantly expressed by microglia and endothelial cells in ALS patients.

Based on this striking pattern of expression of α5 integrin in the motor system in ALS, we validated a potential therapeutic role of anti-α5 integrin antibody in SOD1^G93A^ transgenic mice by treating them with an anti-α5 integrin antibody. The anti-α5 integrin treatment extended survival, improved motor symptoms, and mitigated proinflammatory responses, suggesting that α5 integrin may be a potential therapeutic target for modulation of neuroinflammation in ALS.

## Results

### α5 Integrin Expression Is Associated with Severe Pathology and Myeloid Cells with Distinct Proinflammatory Properties in SOD1^G93A^ ALS Mice.

**Fig. 1.**
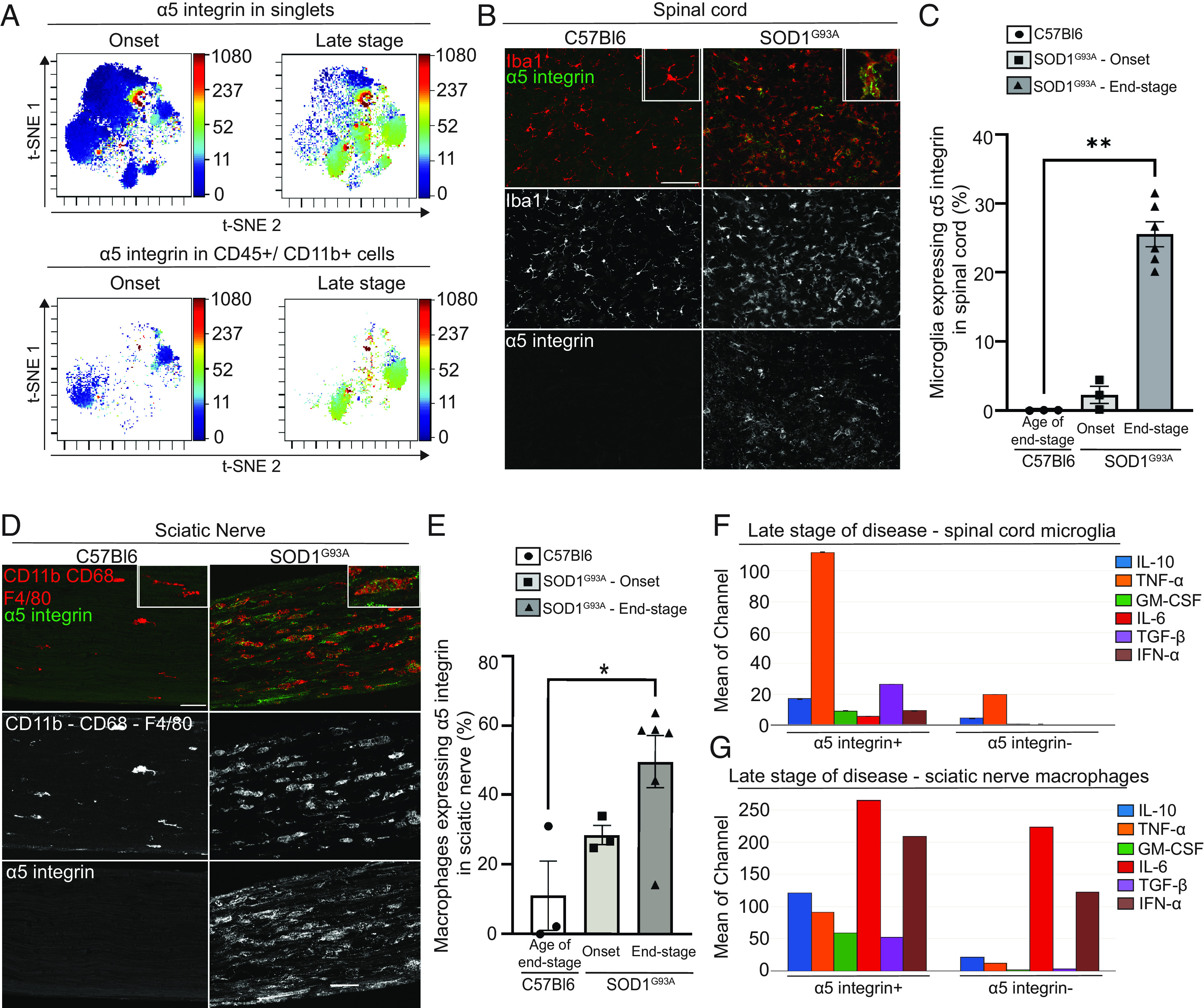
Microglial cells and sciatic nerve macrophages express α5 integrin in ALS mice and display a highly inflammatory profile. (*A*) t-SNE clustering showing α5 integrin expression in the whole single-cell population (n = 78,293 cells, *Upper*) and in CD45+/CD11b+ cells (n = 21,250 cells, *Lower*). (*B*) Spinal cord sections of control (C57Bl6) and SOD1^G93A^ mice at the age of end stage and disease end stage, respectively (on average 164 d) stained for α5 integrin (green) and the microglial cell marker Iba1 (red). (Scale bar, 50 μm.) (*C*) Proportion of spinal cord microglial cells (Iba1+) expressing α5 integrin in percent. n = 3 (C57Bl6 and SOD1^G93A^ – onset), n = 6 (SOD1^G93A^—end stage) mice. Data are shown as means ± SEM (*D*) Sciatic nerve sections of control (C57Bl6) and SOD1^G93A^ mice at the age of end stage and disease end stage, respectively (on average 164 d) stained for α5 integrin (green) and macrophage markers CD11b-CD68 and F4/80 (red). (Scale bar, 50 μm.) (*E*) Proportion of sciatic nerve macrophages (Cd11b-CD68-F4/80 positive) expressing α5 integrin in percent. n = 3 (C57Bl6 and SOD1^G93A^—onset), n=6 (SOD1^G93A^—end stage) mice. Data are shown as means ± SEM. (*F* and *G*), Measure of cytokine expression (IL-10, TNF-α, GM-CSF, IL-6, TGF-β, and IFN-α) in spinal cord microglial cells expressing α5 integrin or not (*F*) and in sciatic nerve macrophages expressing α5 integrin or not (*G*) by CyTOF. Cytokine expression is expressed in mean of channel. n = 10 to 12 pooled mice in seven experiments (*F*), n = 3 pooled mice in one experiment (*G*). *P < 0.05, **P < 0.01 determined by one-way ANOVA (Kruskal–Wallis test) followed by a multiple comparison (*C* and *E*).

Our recent study using CyTOF as a high-throughput and high-resolution, single-cell proteomic screening platform identified elevated α5 integrin on myeloid cells, but not on T or B cells in experimental autoimmune encephalomyelitis (EAE), a neuroinflammatory model that shares some features with multiple sclerosis (22). In the current study, we extended these analyses and evaluated α5 integrin expression in 78,293 single cells, including 21,250 CD45^+^/CD11b^+^ myeloid cells from CNS of SOD1^G93A^ ALS mice, using an unbiased computational approach. Unsupervised clustering with T-stochastic neighbor embedding (t-SNE) revealed subpopulations of CD45^+^/CD11b^+^ CNS myeloid cells expressing α5 integrin in the late stages of motor neuron disease (defined by severe paralysis at 140 d) compared to disease onset (95 d) (Fig. 1*A*). Immunohistological analysis of the spinal cord, the primary site of pathology in SOD1^G93A^ mice, confirmed increased expression of α5 integrin at disease end-stage in SOD1^G93A^ mice compared to age-matched wild-type (WT) mice (Fig. 1*B*). Consistent with our single-cell CyTOF results showing that alpha5 integrin was expressed by myeloid cells, α5 integrin staining was mostly colocalized with ionized calcium-binding adaptor molecule 1 (Iba1), a pan-macrophage marker expressed by microglial cells in the CNS, suggesting that they are the main cell type expressing this integrin in the late stage of this disease model (Fig. 1*B*). Quantification of microglial cells (DAPI^+^, Iba1^+^) expressing α5 integrin in the ventral horn of the spinal cord revealed α5 integrin was significantly increased in SOD1^G93A^ end-stage mice (25.6 ± 1.8%) compared with C57Bl6/J control mice (0.06 ± 0.03%) (Fig. 1*C*). We recently showed the pathophysiological importance of peripheral macrophages in the sciatic nerve of ALS SOD1^G93A^ mice and patients (8). Therefore, we compared α5 integrin expression in the sciatic nerve of SOD1^G93A^ mice with that of WT mice. The expression of α5 integrin in the sciatic nerve was increased in SOD1^G93A^ mice, predominantly at the disease end-stage (Fig. 1*D*). Costaining of α5 integrin with a cocktail of myeloid cell markers (CD11b, CD68, and F4/80) revealed increased α5 integrin expression in sciatic nerve macrophages (49.6 ± 7.6%) at end stage compared to control mouse sciatic nerve macrophages (11.07 ± 10%) (Fig. 1*E*). To determine whether the myeloid cells expressing α5 integrin in the spinal cord and sciatic nerve of SOD1^G93A^ mice had proinflammatory properties, which may play a role in the pathophysiology of ALS, we analyzed cytokine expression by CyTOF at disease end-stage (*SI Appendix*, Tables S1 and S2). In the spinal cord, α5 integrin–positive myeloid cells produced significantly higher levels of all cytokines measured––TNF-α, GM-CSF, IL-6, IFN-α, TGF-β, and IL-10 compared to α5 integrin–negative or low expressing microglia (Fig. 1*F*). TNF-α, a highly proinflammatory cytokine, was significantly up-regulated (fivefold increase) in α5 integrin–positive CNS myeloid cells (Fig. 1*F*). Similarly, sciatic nerve macrophages produced higher levels of cytokines compared to α5 integrin–negative macrophages (Fig. 1*G*). IL-6 and TGF-β were the most prominent cytokines produced by α5 integrin–positive macrophages (Fig. 1*G*). To determine whether our findings were consistent with results based upon different methodologies, we mined previously published transcriptomic datasets of SOD1^G93A^ microglial cells from two studies (8, 11) and confirmed increased α5 integrin expression in microglial cells (*SI Appendix*, Fig. S1 *A* and *B*). These findings suggest that elevated α5 integrin on myeloid cells may play a role in the pathology of ALS.

### Increased Expression of α5 Integrin in the Ventral Horn and Motor Tracts in ALS with Different Clinical Presentations and Disease Durations.

To further explore the significance of α5 integrin in ALS, we investigated the presence of α5 integrin and colocation with microglia in ALS patients with a range of clinical presentations and disease durations. We studied autopsy tissues from 132 ALS patients. Most had abnormal TDP-43 aggregates, including patients with sporadic ALS and c9ALS, as well as ALS patients with rare genetic variants. The latter included familial ALS due to mutations in *TARDBP* and ALS associated with mutations in *TBK1 or* *SOD1*.

Since adult-onset ALS is characterized by selective loss of motor neurons, especially the large α-motor neurons in the ventral horn of the spinal cord, we assessed neuronophagia with Iba1 immunohistochemistry in 132 ALS patients. Markedly increased Iba1-positive microglia were detected in 99 cases (75%) using a semi-quantitative approach (0 = none, 1 = mild, 2 = moderate, and 3 = marked). Immunoreactive microglia had morphological features of activated microglia, with amoeboid rather than ramified morphology. There were also activated microglia in the lateral and anterior corticospinal tracts. Neuronophagia was assessed in anterior horns as an indicator of active motor neuron disease. Neuronophagia was defined as clusters of microglial cells in the ventral horn often with an “empty cell bed” where a degenerating neuron had resided. Neuronophagia was detected in cervicothoracic, thoracic, and lumbar sections of the spinal cord in 18 of 132 patients, irrespective of etiology (sporadic, *C9ORF72, SOD1, TARDBP,* and *TBK1*) (*SI Appendix*, Table S3). We compared ALS patients with active neuronophagia to individuals without motor neuron disease (N = 10).

**Fig. 2.**
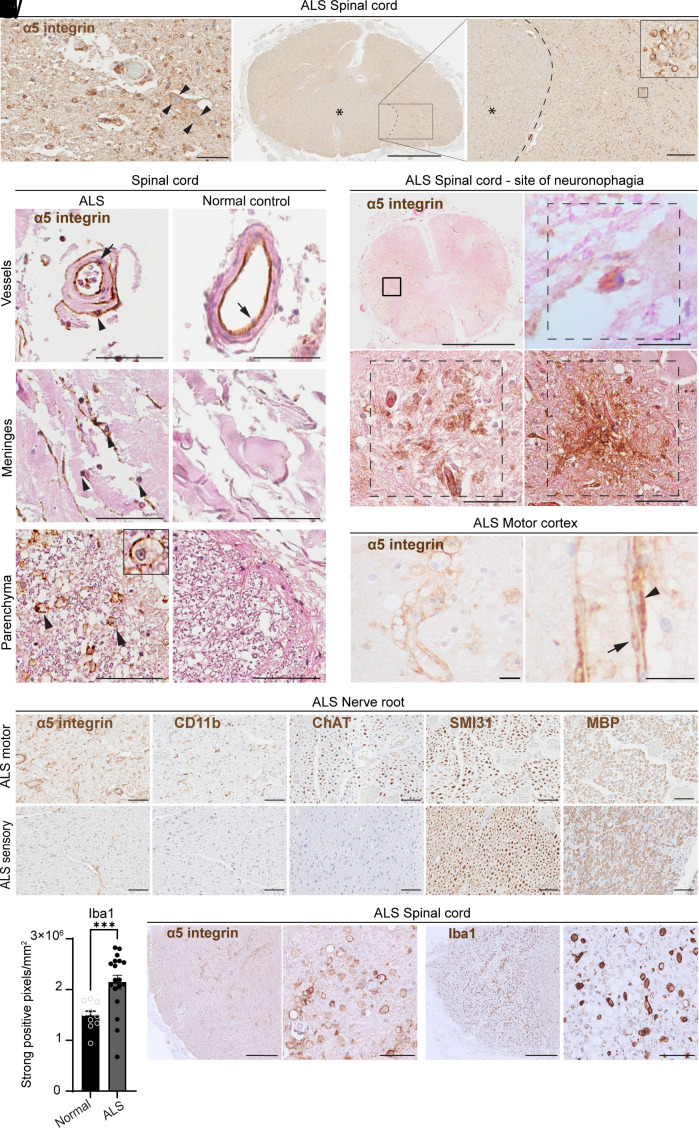
Expression of α5 integrin is specific to ALS and restricted to motor areas. (*A*–*C*) Spinal cord section of an ALS case showing α5 integrin expression in the ventral horn, including vasculature and neurons (arrowheads), Scale bar: 60 μm (*A*). A spinal cord whole section shows increased α5 integrin in the lateral corticospinal tract microglia (boxed area and *Inset*), compared to the dorsal columns (*) (*B* and *C*) Scale bars: 2 mm (*B*), 300 μm (*C*). (*D*–*I*) Increased expression of α5 integrin in spinal cord vessels highlights α5 integrin–positive endothelial cells (arrow) in ALS and normal controls, but only ALS demonstrates α5 integrin–positive perivascular cells (arrowheads) (*D* and *E*), meningeal cells (arrowheads) (*F* and *G*), and parenchymal foamy macrophages (arrowheads) (*H* and *I*); ALS (*D*, *F*, and *H*), control (*E*, *G*, and *I*). Scale bars: 60 μm (*D*–*G*), 80 μm (*H* and *I*). (*J*–*M*) Expression of α5 integrin in the spinal cord ventral horn shows various phases of neuronophagia: α5 integrin–positive cell in juxtaposition to a large motor neuron (*K*), α5 integrin–positive microglial cell cluster and vessels at the site of a previous neuron “empty cell bed” (*L*), α5 integrin–positive cells in the scarred ventral horn (*M*), Scale bars: 3 mm (*J*), 20 μm (*K*), 50 μm (*L*), 80 μm (*M*). (*N* and *O*) α5 integrin expression in the motor cortex of an ALS case showing α5 integrin–positive endothelial cells (arrow, *O*), perivascular cells (fibroblasts or pericytes—arrowhead, *O*), and α5 integrin–positive histiocytes in ALS (*N*). (*P*–*T*) Peripheral ALS motor nerve showing moderately increased α5 integrin (*P*) and mildly increased CD11b (*Q*). Surviving motor fibers stain positive for axon marker ChAT (*R*), SMI31 (*S*), and for myelin marker MBP (*T*). Axon marker SMI31 and myelin marker MBP are decreased compared to ALS sensory nerve (*S* and *T*), sensory fibers are distinguished by their lack of ChAT staining (*R*). (Scale bars: 60 μm.) (*U*) Quantification of Iba1 staining in the spinal cord of ALS patients compared to normal individuals showing increased Iba1 expression in ALS. ***P < 0.001 determined by *t*-test, n = 10 for normal control individuals and n = 18 for ALS. (*V*–*Y*) Expression of α5 integrin (*V* and *W*) and Iba1 (*X* and *Y*) in ALS spinal cord sections showing that the majority of α5 integrin–positive cells are Iba1 positive. Scale bars: 500 μm (*V* and *X*) and 60 μm (*W* and *Y*).

We found higher expression of α5 integrin in ALS compared to normal controls in the lateral corticospinal tracts and ventral horns, irrespective of the etiology of ALS or the disease duration (Fig. 2 *A–C*). The expression of α5 integrin was detected in cells surrounding motor neurons in the ventral horn (Fig. 2*A*). In addition, endothelial cells in spinal cord vessels and meninges of ALS patients had higher expression of α5 integrin compared to normal controls (Fig. 2 *D–I*). Importantly, α5 integrin was increased not only in cells adjacent to ventral horn neurons (Fig. 2*H*), but also in “empty cell beds” and areas of gliosis in the anterior horn (Fig. 2 *J–M*). A subset of ALS patients with severe motor cortex involvement had increased α5 integrin immunoreactivity in the parenchyma and blood vessels, sometimes associated with foamy macrophages, a microglial morphology associated with a phagocytosis (Fig. 2 *N* and *O*).

Similar selective involvement of motor tracts was also evident in nerve roots from ALS patients. Expression of α5 integrin was detected in anterior roots, which contain motor neuron axons, but not in posterior roots and dorsal root ganglion where sensory neuronal axons originate (Fig. 2 *P*–*T*).

We further confirmed elevated α5 integrin expression in ALS by mining previously published transcriptomic data from spinal cord ventral horns of postmortem ALS human donors (23). This analysis confirmed that α5 integrin expression is significantly increased in the spinal cord of ALS patients compared to controls (*SI Appendix*, Fig. S1*C*).

Our CyTOF analysis and the gene expression analyses from other groups have shown that microglia and sciatic nerve macrophages express elevated levels of α5 integrin in the SOD^G93A^ ALS mouse model. Thus, we further evaluated the expression of Iba1 (microglial marker) and its correlation with α5 integrin in the spinal cord of ALS patients and patients without MND. Consistent with previous studies, Iba1 was significantly increased in the ALS spinal cord compared to normal controls (Fig. 2*U*). Foamy macrophages and α5 integrin–positive cells were increased in the same areas in ALS (Fig. 2 *V*–*X*). Together, detailed analysis of α5 integrin staining in ALS revealed prominent expression of α5 integrin by microglia in affected motor regions. These findings were not observed in normal controls.

### α5 Integrin Expression Is Increased Irrespective of the ALS Subtype and Duration of the Disease.

**Fig. 3.**
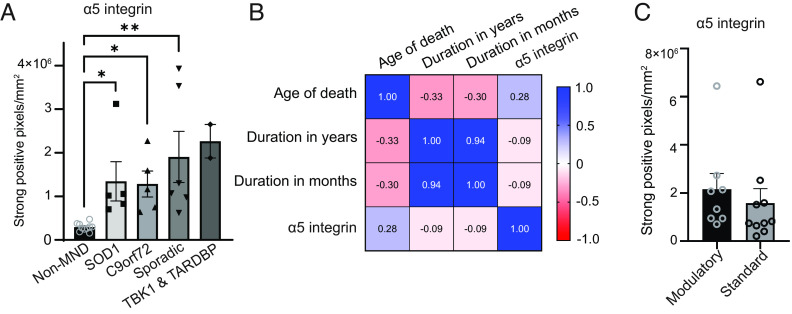
Expression of α5 integrin is increased in ALS irrespective of disease etiology and is not targeted by available treatments. (*A*) Quantification of α5 integrin expression in the spinal cord from normal control individuals (n = 10), SOD1 ALS cases (n = 5), C9orf72 ALS cases (n = 5), sporadic ALS cases (n = 6), and TBK1 (n = 1) and TARDBP (n = 1) ALS cases grouped together. *P < 0.05 and **P < 0.001 determined by one-way ANOVA followed by a multiple comparison. (*B*) Correlation matrix comparing α5 integrin expression with age of death and duration of disease in years and in months (n = 18 ALS cases). (*C*) Quantification of α5 integrin expression in the spinal cord of ALS patients receiving standard treatment (no treatment or riluzole only, n = 8), or modulatory treatment (prednisone, TNF-α, edaravone, antisense oligonucleotide, mesenchymal stem cell therapy, n = 10).

Immunohistological assessment showed a significant increase of α5 integrin expression in ALS patients compared to normal controls (Fig. 3*A*). More importantly, a subanalysis across ALS etiologies confirmed increased expression of α5 integrin in the spinal cord compared to controls irrespective of ALS subtype (Fig. 3*A*). Furthermore, α5 integrin was significantly increased in the ALS spinal cord regardless of age of death or disease duration (Fig. 3*B*). We next used CyTOF to examine α5 integrin–positive myeloid cells in an additional cohort of ALS patients (*SI Appendix*, Fig. S2*A* and Table S5). α5 integrin– positive cells represented more than 40% of the myeloid cells (CD11b+) in ALS patient cervical spinal cord (48.5 ± 10.8%, n = 3) and motor cortex (42.8 ± 4.9%, n = 3), without significant differences between the two regions (*SI Appendix*, Fig. S2 *A* and *B*). Additional CyTOF analysis revealed that α5 integrin–positive cells expressed higher levels of HLA-DR and CD74, critical for antigen presentation, in addition to CD11c and CD9 compared to α5 integrin–negative myeloid cells (*SI Appendix*, Fig. S2*C*). Interestingly, these markers are part of the signature of disease-associated microglia (DAM), a subgroup of microglial cells primarily associated with neurodegeneration (24, 25). Addressing whether the clinical treatments that ALS patients received might have mitigated α5 integrin expression, we performed an analysis after subgrouping patients into two treatment groups to compare patients that received no treatment or riluzole only (labeled “Standard” in Fig. 3*C*), to patients that received drugs such as prednisone, TNF-α, edaravone, antisense oligonucleotide, or mesenchymal stem cell therapy (labeled “Modulatory” in Fig. 3*C*). There was no significant difference in α5 integrin in the spinal cord between the two groups, highlighting that α5 integrin is not adequately targeted by current modulatory treatment strategies.

### α5 Integrin Demonstrates Specificity to ALS Pathology Compared to Another Druggable Integrin.

To address the specificity and the distribution pattern of α5 integrin in ALS compared to other integrins, we stained the ALS spinal cord for α5 integrin and compared findings to another druggable integrin, α4. In 1992, we identified α4 integrin as the key molecule involved in homing of leukocytes to inflamed regions of the brain (26). An antibody to α4 integrin called natalizumab has been approved as a therapy for multiple sclerosis (26). Recently, a study demonstrated that treating SOD1^G93A^ and TDP43^A315T^ mice, another ALS model, with natalizumab prolonged survival in these models (27). Since natalizumab blocks homing of all leukocytes, it has been shown to be associated with progressive multifocal leukoencephalopathy in JC virus–seropositive patients by preventing lymphocytes from adhering to the endothelium of the blood–brain barrier, thereby reducing their migration from the blood into the CNS and suppressing T cell–mediated immune responses in the brain (28). To determine whether α5 integrin might present a better druggable target in ALS compared to the α4 integrin, we compared spinal cord expression of α5 integrin versus α4 integrin in ALS (*SI Appendix*, Fig. S3 *A* and *B*). In contrast to α5 integrin, α4 integrin was not expressed by foamy macrophages in ALS (*SI Appendix*, Fig. S3*B*). Myelin staining with Luxol fast blue suggested that α4 integrin was expressed in the myelin of ALS patients (*SI Appendix*, Fig. S3*A*). ALS spinal cord tracts demonstrating myelin pallor had fragmented α4 integrin myelin profiles in the ventral horn (*SI Appendix*, Fig. S3 *A* and *B*) and in the lateral corticospinal tract (*SI Appendix*, Fig. S3*B*), including cells morphologically consistent with oligodendrocytes (*SI Appendix*, Fig. S3*B*, arrowheads). Lack of α4 integrin immunoreactivity in microglia in ALS patients indicates that α4 integrin is unlikely to contribute to neuronophagia or tissue destruction in ALS.

### Anti-α5 Integrin Has Therapeutic Potential by Improving Motor Symptoms and Increasing Survival in a Preclinical Model of ALS.

**Fig. 4.**
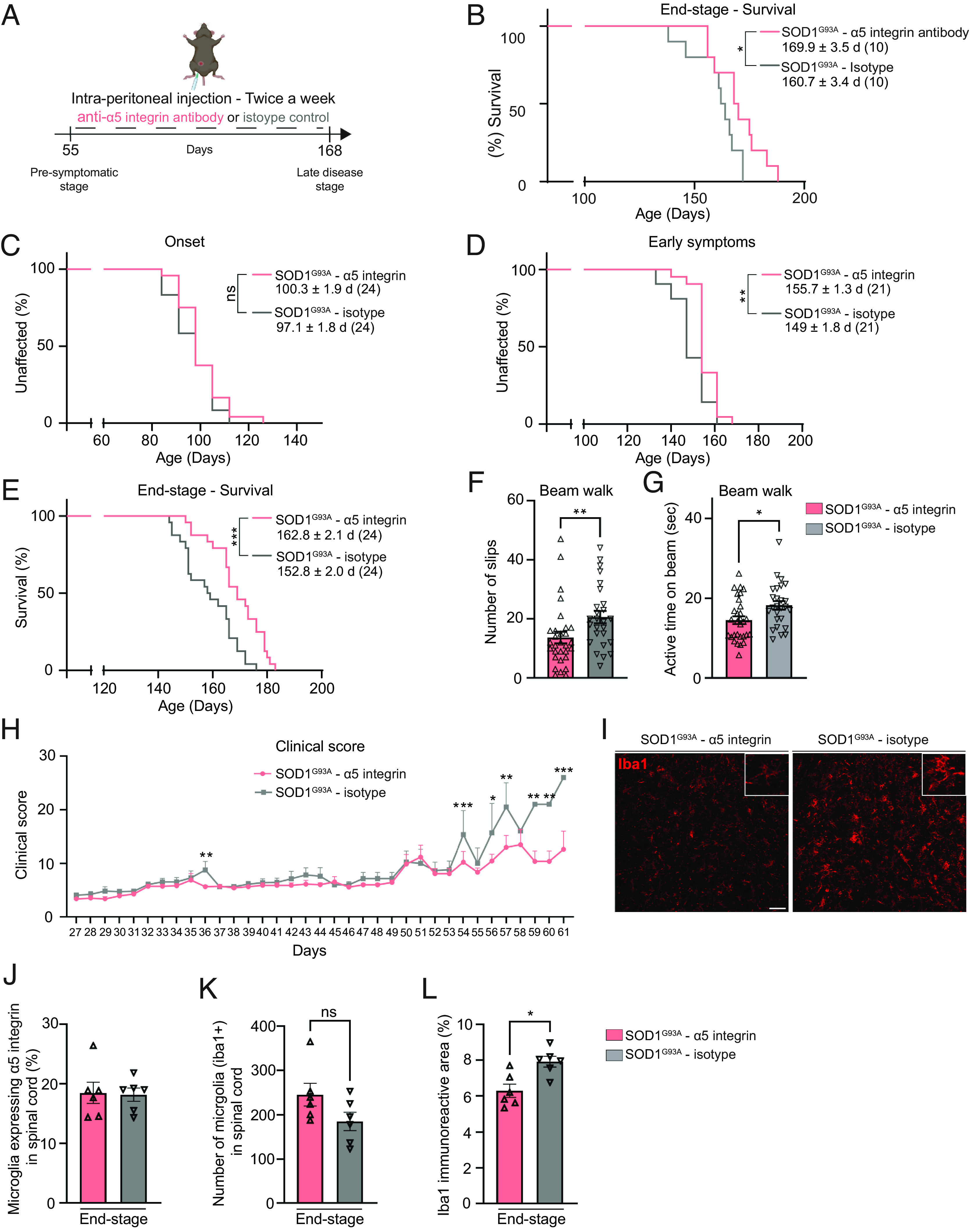
Anti-α5 integrin treatment improves motor functions and survival of SOD1^G93A^ ALS mice. (*A*) Protocol used to treat SOD1^G93A^ mice with anti-α5 integrin antibody or isotype control. (*B*) Kaplan–Meier plot of ages reached at disease end-stage (complete hindlimb paralysis) in SOD1^G93A^ mice injected with anti-α5 integrin (pink) or isotype control (gray). (*C*–*E*) Kaplan–Meier plot of ages reached at disease onset [defined by the peak of weight (*C*)], early symptomatic stage [defined by the 10% of weight loss (*D*)], or end-stage [complete hindlimb paralysis (*E*)] in SOD1^G93A^ mice injected with anti-α5 integrin (pink) or isotype control (gray). Legend shows mean ages ± SEM with the number of mice in parentheses. (*F* and *G*) Motor balance assessment by the beam walk test: number of slips (*F*) and active time on the beam (*G*) for SOD1^G93A^ mice injected with anti-α5 integrin (pink, n = 30) or isotype control (gray, n = 26). Mice falling off the beam were excluded: n = 4 in isotype control–treated group. Data are shown as means ± SEM (*H*). Clinical score assessed daily for SOD1^G93A^ mice injected with anti-α5 integrin (pink) or isotype control (gray) from 139 days-old (27 d after the start of the clinical score assessment) to 173 days-old (61 d after the start of the clinical assessment). Score 0: normal mouse, to score 20: complete paralysis, see Material and Methods for detailed scoring. n = 30 per group until 141 d; after that, the number of mice decreases as they reach end stage. Data are shown as means ± SEM (*I*), Spinal cord sections of SOD1^G93A^ mice treated with anti-α5 integrin at end stage stained for microglial cell marker Iba1 (red). (Scale bar, 50 μm.) (*J*–*L*) α5 integrin expression and microglial cell reactivity: count of spinal cord microglial cells (Iba1 and DAPI positive) expressing α5 integrin in percent (*J*), count of microglial cells (Iba1 and DAPI positive, (*K*)) and measure of Iba1-immunoreactive area (*L*). n = 6 for both groups (*J*–*L*). Data are shown as means ± SEM *P < 0.05, **P < 0.01, ***P < 0.001 determined by log-rank test (*B*–*E*), Mann–Whitney *U* test (*F*, *G*, *K*, and *L*) and mixed-effect analysis and Bonferroni’s multiple comparison test (*H*).

To determine the therapeutic potential of blocking α5 integrin in ALS, we explored the potential effect of modulating α5 integrin expression in the SOD1^G93A^ ALS mouse model. Despite the discovery of numerous additional genes related to the genetic forms of ALS disease, SOD1^G93A^ mice are still the most dependable model in developing an ALS-like phenotype. This model can be used to measure the outcome of potential ALS treatments by assessment of parameters of disease progression, including age at onset, lifespan, disease duration, inflammation, and motor performance. In a pilot study, we treated a small cohort of SOD1^G93A^ mice with anti-α5 integrin antibody, MFR5, which blocks adhesion of α5 integrin–expressing cells. A group of 10 SOD1^G93A^ mice received intraperitoneal (IP) injections of MFR5 two times per week and 10 mice received the isotype control antibody (Fig. 4*A*). Blocking α5 integrin in SOD1^G93A^ mice extended the survival of mice (169.9 ± 3.5 d) compared to isotype controls (160.7 ± 3.4 d) (Fig. 4*B*). These data show that α5 integrin plays a role in the pathology of ALS and identifies a previously unreported therapeutic target. To determine the impact of blocking α5 integrin on improving motor symptoms and to determine whether our pilot study was reproducible when performed in another laboratory (a useful standard in experiments including animal models), we tested the effect of long-term treatment of a larger cohort of SOD1^G93A^ and control mice (N = 60) at QPS Neuropharmacology in Austria. Prior to the validation cohort study, we performed pharmacokinetic (PK) and receptor occupancy (RO) studies in a group of control mice (C57Bl/6) to understand whether the dosing regimen of biweekly injections of anti-α5 integrin antibody, as performed in the preliminary study, was sufficient to block α5 integrin in the peripheral circulation and CNS. After a single IP injection at 5 mg/kg, the anti-α5 integrin (MFR5) antibody was measured in blood and cerebrospinal fluid (CSF), and receptor occupancy was determined on blood cells (*SI Appendix*, Fig. S4 *A* and *B*). The results of the PK study show that serum anti-α5 integrin antibody reached Cmax at 4 h at approximately 100 µg/mL, and at 72 h was at 31.5 µg/mL (*SI Appendix*, Fig. S4*A*). Anti-α5 integrin antibody concentrations in CSF at 24 and 72 h after the single injection were approximately 1,000-fold lower (0.1%) than what was measured in serum at the same time points (*SI Appendix*, Fig. S4*A*), indicating that low levels of this antibody penetrate the blood–brain barrier, which is typical of what is seen with IgG antibodies in general (2). The results of the RO study show that after a 5-mg/kg IP injection of the anti-α5 integrin antibody, monocytes and NK cells have greater than 90% RO out to 72 h (*SI Appendix*, Fig. S4 *B* and *C*). Notably, the anti-α5 integrin antibody was not significantly detected in B and T cells, which is consistent with the lack of α5 integrin expression in these cell types, and consistent with what we reported previously (22). The PK and RO data taken together suggest that biweekly injections of the anti-α5 integrin antibody at 5 mg/kg, as was used in the pilot study, are sufficient to achieve high levels of exposure in peripheral circulation and low levels of exposure in the CNS. Thus, the biweekly dosing scheme was continued for the larger validation study performed at QPS Neuropharmacology.

Survival, behavioral, and histological outcomes of treatment were measured in SOD1^G93A^ mice that received either the anti-α5 integrin antibody (MFR5; n = 30) or an isotype control antibody (n = 30) by IP biweekly injections starting at 8 wk until disease end-stage or until 20 wk (late symptomatic stage) when tissue was harvested (n = 6 per group). In-depth analysis of the disease course of anti-α5 integrin–treated ALS mice revealed a similar age of disease onset, but a delay of the symptomatic stage and a longer survival compared to isotype-treated ALS mice (Fig. 4 *C–E*), consistent with microglia/macrophages playing a major role in ALS disease progression but not in its initiation (3, 4). These results show that α5 integrin expression is strongly associated with disease progression in SOD1^G93A^ mice (Fig. 4).

To assess the effect of anti-α5 integrin treatment on motor phenotype, we performed several behavioral analyses. The beam walking test, a measure of coordination and balance, revealed significantly fewer slips (Fig. 4*F* and *SI Appendix*, Fig. S4*D*) and reduced latency to traverse the beam (Fig. 4*G*) in the group treated with anti-α5 integrin antibody compared to the isotype-treated group. Similarly, Vercelli scoring, used to assess motor phenotype in ALS mice (29), showed a trend toward better scores for α5 integrin–treated mice, with a significant difference at week 18 (corresponding to early disease stage) compared to isotype-treated mice (*SI Appendix*, Fig. S4*E*). Finally, a general evaluation of ALS mouse phenotype and health assessed daily, from week 16 (corresponding to early disease stage and considered as day 1 in the *SI Appendix* graph) until disease end stage, revealed significantly lower scores during the symptomatic phase of the disease, indicative of better general health and reduced symptoms, in α5 integrin–treated ALS mice compared to isotype-treated mice (Fig. 4*H* and *SI Appendix*, Fig. S4*H*).

In the current study, we have shown using CyTOF, transcriptomic profiling, and immunohistochemistry that microglia are the main immune cells expressing α5 integrin in ALS. Microglia are known to become activated in response to motor neuron degeneration (Fig. 2).

Therefore, we analyzed the effect of anti-α5 integrin treatment on microglial cells and Iba1 immunoreactivity, in the spinal cords of SOD1^G93A^ mice (Fig. 4 *I–L*). While the percent of microglial cells expressing α5 integrin (Fig. 4*J*) and the number of microglial cells (Fig. 4*K*) remained unchanged, the Iba1 immunoreactivity was significantly decreased in α5 integrin–treated SOD1^G93A^ mice compared to isotype-treated mice (Fig. 4 *I* and *L*). Note that the immunoreactive area measurement considers both the surface occupied by the staining and its intensity. As microglial activation is associated with larger cell body size and an increase of Iba1 intensity, a decrease in both parameters may indicate lower microglial reactivity in the anti-α5 integrin–treated mouse cohort compared to isotype-treated mice. The findings suggest that mitigation of an α5 proinflammatory microglial response may contribute to increased SOD1^G93A^ mouse survival.

## Discussion

Pathology in ALS and in the SOD1^G93A^ model is accompanied by robust activation of microglia, which are considered to be involved in disease progression (9, 10, 12, 30). Our recent study highlighted that peripheral nerve macrophages also play a role in aggravating motor neuron degeneration in ALS (8). The causative role of inflammation, particularly microglia or peripheral nerve macrophages, in ALS pathology has been the subject of intense investigation (31). A major barrier in elucidating the molecular mechanisms of inflammation-induced ALS pathology has been the heterogeneity of microglia, underscoring the need for single-cell studies. By utilizing CyTOF, a single-cell proteomics platform, we identified that α5 integrin was expressed on microglia and peripheral nerve macrophages in SOD1^G93A^ ALS mice. We further identified a significant increase of α5 integrin on microglia in ALS spinal cords compared to normal controls and to other neurodegenerative disorders. We demonstrated that targeting α5 integrin with anti-α5 integrin antibody extended survival and improved motor performance in SOD1^G93A^ ALS mice.

Our single-cell analysis of spinal cord microglia and peripheral nerve macrophages revealed a significant upregulation of α5 integrin as the disease progressed to the end stage. The α5 integrin–positive myeloid cells demonstrated a proinflammatory phenotype and produced significantly higher levels of all proinflammatory cytokines, particularly TNF-α (in spinal cord microglia) and IL-6 (in peripheral nerve macrophages), compared to α5-negative or low expressing cells. One of our recent studies demonstrated that infiltration and activation of peripheral nerve macrophages has a role in exacerbating motor neuron degeneration in SOD1^G93A^ ALS mice (8). Here, we show that α5 integrin–positive macrophages produced higher levels of the IL-6 cytokine, which is known to participate in macrophage recruitment toward neurons (32, 33). In parallel, α5 integrin–positive peripheral nerve macrophages also highly up-regulated TGF-β, a nerve growth factor known to mediate the expression of extracellular matrix proteins such as fibronectin, a primary ligand of α5 integrin (34). Considering that α5 integrin–positive myeloid cells express proteins tightly linked to macrophage recruitment and migration, we propose that the presence of α5 integrin–positive macrophages in the peripheral nerve may participate in further recruitment of additional macrophages to the nerve, exacerbating inflammation and axonal degeneration. It is intriguing that primary motor neurons and motor nerve roots as they leave the central nervous system selectively express α5 integrin compared to primary sensory neurons and sensory nerve bundles (Fig. 2 *L–P*). This implies that even in the periphery there is spatial zonation of α5 integrin between motor and sensory systems, consistent with clinical findings of preserved sensory neurons in ALS.

In situ analysis of human postmortem tissues revealed that α5 integrin–positive microglia were significantly increased in human ALS postmortem nerve fibers. This increase was selective to motor nerves and associated with a decrease in motor nerve fibers compared to sensory nerve fibers. In addition, in the ALS spinal cord, α5 integrin expression was also increased across sporadic and genetic (*C9orf72, SOD1, TARDBP,* and *TBK1*) ALS subtypes compared to non-MND controls. Interestingly, additional CyTOF analysis of postmortem human CNS myeloid cells revealed that α5 integrin–positive cells overexpressed several markers of the DAM signature. While this signature has been primarily identified in mouse models of Alzheimer’s disease and ALS, human studies have only been able to identify partial signatures in several neurodegenerative conditions so far (24, 25). Nevertheless, DAM cells are known to be metabolically active expressing genes involved in various functions, including phagocytosis, lysosomal functions, and tissue remodeling (25). Interestingly, we have observed the presence of α5 integrin–positive microglia, particularly in the zone of active and previous neuronophagia of the anterior horn of the postmortem ALS patient spinal cord. Altogether, this suggests a major role of α5 integrin in ongoing motor neuron damage.

Importantly, findings from postmortem tissue studies show that ALS patients irrespective of sex, age, disease duration, genotype, or treatment consistently had increased α5 integrin–positive microglial cells, specifically in damaged motor areas, suggesting that modulation of α5 integrin–positive immune cells may prove beneficial for ALS across a wide range of ALS etiologies. Comparing the distribution of α5 to α4 integrin, another α-integrin involved in focal adhesion that has been implicated in experimental animal models of ALS (27), we showed that none of the ALS patients demonstrated α4 integrin–positive microglial cells. This indicates that α4 integrin is unlikely to contribute to neuronophagia or tissue destruction to any significant degree in ALS. These findings suggest that α5 integrin responds to yet unidentified disease-specific cues that likely involve microglial activation. Our findings demonstrate a link between α5 integrin and the pathobiology of ALS and make α5 integrin an attractive candidate for therapeutic modulation in ALS.

Treatment with α5 integrin antibody improved motor functions of SOD1^G93A^ mice as assessed by beam walk assay and Vercelli scoring. In addition, clinical scores assessing general and specific motor deficits related to SOD1^G93A^ ALS mice were lower in α5 integrin–treated mice. This reflects a better overall condition in the α5 integrin–treated mice. Improvement in motor function after treatment with anti-α5 integrin suggests that treatment protects motor neurons from degeneration, which leads to better motor performance. Although we did not detect any difference in Iba1-positive microglia and the number of cells expressing α5 integrin in the spinal cord of mice treated with α5 integrin antibody compared to the isotype control, the decrease in Iba1 immunoreactivity suggests that anti-α5 integrin treatment may have played a role in mitigating neuroinflammation in ALS mice. Anti-α5 integrin antibody concentration was significantly lower in the CNS than that in the blood, which could limit the direct effect of the anti-α5 integrin on microglial cells. However, it has been shown that modulating blood monocytes and macrophages at the periphery can also induce significant changes in microglial cell transcriptomic profiles toward a more neurotrophic phenotype in ALS mice (8, 35). Additionally, while we have shown that circulating blood monocytes do not infiltrate the spinal cord of SOD1^G93A^ ALS mice during disease (8, 20), additional routes of possible monocyte migration from the skull and vertebral bone marrow have recently been identified following CNS inflammation (36). Considering the known role of α5 integrin in leukocyte recruitment and adhesion, targeting α5 integrin could impair the migration of monocytes from the vertebral bone marrow if such a phenomenon would apply to ALS (37). Therefore, the anti-α5 integrin treatment’s effect on motor neuron protection can be due to tempering both peripheral and CNS inflammation by influencing microglial cells directly and indirectly through the modulation of peripheral monocytes and macrophages in ALS.

Our combined findings in this study involving single-cell labeling using CyTOF, transcriptomic profiling, human postmortem ALS tissue studies, and a mouse model of motor neuron disease identified α5 integrin as a potential therapeutic target in ALS patients. We show that α5 integrin upregulation is a common feature of both CNS microglial cells and sciatic nerve macrophages in ALS. By modulating α5 integrin, we could target both cell populations and mitigate a proinflammatory response that may be needed to protect motor neurons from degeneration as well as axonal degeneration in the periphery. These findings provide a molecular link between inflammation and the pathobiology of ALS and provide a promising strategy for clinical application in ALS. There are five approved drugs targeting integrins for different diseases. A clinical trial to test α5 integrin as a drug target in ALS might be worthwhile.

## Material and Methods

### Mice.

For the purpose of this study, SOD1 transgenic mouse lines were used: hSOD1^G93A^ mice [B6.Cg Tg (SOD1*G93A)1Gur/J; #004435, Jackson Laboratory]. Mice carrying the human *SOD1* transgene were identified by PCR screening of tail DNA. C57bl/6J (#000664, Jackson Laboratory) nontransgenic mice were used as controls for histology experiments. Mouse experiments were carried out in three different facilities: Stanford University for mice used in Fig. 1 *A* and *F*, *Upper*, Fig. 4 *A* and *B*; Oregon Health and Science University for mice used in Fig. 1 *B–E* and *F*, *Lower*; and QPS (Austria GmbH) for mice used in Fig. 4 *C–L*. Mouse experiments at Stanford University were approved by the Institutional Animal Care and Use Committee at Stanford University and performed in compliance with HIH guidelines. The QPS (Austria GmbH) animal facility is fully accredited by the Association for Assessment and Accreditation of Laboratory Animal Care (AAALAC). All the procedures in this study comply with the animal care and welfare committee and are in accordance with standard operation procedures. Additional mouse experiments for histology analysis were approved by and performed in compliance with the NIH guidelines of the Institutional Animal Care and Use Committee at Oregon Health and Science University. In all facilities, the mice were housed in individual ventilated cages on standardized rodent bedding. Each cage contained a maximum of five mice. The room temperature was maintained at 20 to 24 °C and the relative humidity was maintained between 45 and 65%. The animals were housed under a constant light cycle (12 h light/dark). Dried, pelleted standard rodent chow and normal tap water were available to the animals ad libitum.

### Treatment.

#### Small cohort (Fig. 4 *A* and *B*).

Twenty female SOD1^G93A^ mice were randomly divided in two groups of 10 mice. Each group received biweekly intraperitoneal injections of either anti-α5 integrin antibody or isotype control (100 µg) starting at 8 wk old, corresponding to the presymptomatic stage, until the mice reached end stage (on average 169.9 ± 3.5 d for anti-α5 integrin antibody–treated mice and 160.7 ± 3.4 d for isotype control–treated mice). Mouse body weights were assessed weekly. Antibody and isotype were diluted in 0.1M PBS and freshly made every week.

#### Validation cohort (Fig. 4 *C*–*L*).

Sixty female SOD1^G93A^ transgenic mice were randomly assigned to two groups. The first group (Group 1) of 12 animals comprises the in vivo part including treatment of animals, in vivo blood sampling, and behavioral analysis. The second group (Group 2) of 48 animals was used for survival analysis. Among Group 2,12 animals (n=6 per treatment condition) were used for tissue and fluid collection. In each group, half of the mice received either anti-α5 integrin antibody or isotype control. Group 1 received a biweekly treatment per IP injection of either anti-α5 integrin antibody or isotype antibody (4 mg/kg each) starting at an age of 8 wk for a duration of 13 wk. For Group 2, the biweekly treatment was maintained up to 28 wk of age. Anti-α5 integrin antibody (Ultra-LEAF™ Purified rat anti-mouse α5 integrin Antibody, 5H10-27 (MFR5), BioLegend) and isotype control antibody (Ultra-LEAF™ Purified Rat IgG2a, Isotype Control Antibody, RTK2758, BioLegend) solutions were prepared weekly at 4 mg/kg in 0.1M PBS (Lonza). Mouse body weights were assessed weekly, and daily health checks were performed to monitor clinical symptoms starting at 16 wk old.

### Behavioral Assessment.

Vercelli score and beam walk test were performed for all animals to assess the motor behavior of all animals. All animals were examined using the Vercelli score as a reference standard for seven times (week 8—baseline, 10, 12, 14, 16, 18, and 20). Beam walk test (two beams tested) was performed once at an age of 18 wk. The behavioral testing was done on nondosing days. Clinical score was assessed daily starting at 16 wk old. For more details, see **SI Appendix*, Materials and Methods*.

### Mouse Single-Cell Isolation.

#### For microglial cells.

Before anesthesia, mice received an intravenous injection of 100 µL of protein transport inhibitor (eBioscience, 1:500, 00-4980-93) in the tail vein. After that step, every buffer contained the protein transport inhibitor cocktail (1:500) except during the Percoll gradient. After 30 min, the mice were anesthetized with an intraperitoneal injection of ketamine (100 mg/kg). Upon the loss of nociceptive reflexes, the animals were perfused transcardially with ice-cold 0.1M PBS. Brains and spinal cords were removed and gently homogenized in cold 0.1M HBSS (Life Technologies, 14175-095) on ice. Mononuclear cells were separated with 30 to 70% Percoll (GE Healthcare) gradient centrifugation. Cell suspensions were washed in 0.1M PBS with 2% FCS and 2 mM EDTA two times. Cells were resuspended in PBS with 2% FCS and the protein transport inhibitor cocktail (1:500) and incubated on a shaker for 4 h at 37 °C. At the end of the incubation, the cells were directly fixed with 1.6% PFA for 10 min on a shaker at room temperature, washed two times with 0.1M PBS with 2%FCS, and frozen at –80 °C. In each experiment, 10 to 12 mice were pooled to provide enough cells. The experiment was repeated 10 times. Samples from each condition were mass-tag cell barcoded (MCB). In each sample, a unique combination of 6 palladium isotopes was used to encode 20 unique mass-tag barcodes as previously described (22).

#### For sciatic nerve macrophages.

Before anesthesia, mice received an intravenous injection of 100 µL of protein transport inhibitor cocktail (eBioscience, 1:500, 00-4980-93) in the tail vein. After 30 min, the mice were anesthetized with an intraperitoneal injection of ketamine (100 mg/kg). After that step, every buffer contained the protein transport inhibitor cocktail (1:500). The mice were perfused transcardially with 20 mL of ice-cold 0.1M PBS. Both sciatic nerves were removed gently and placed in cold 0.1M HBSS (Life Technologies, 14175-095) on ice. The sciatic nerves were transferred in an Eppendorf tube containing an enzymatic digestion buffer [0.1 M HBSS solution containing 2 mg/mL Dispase II (Sigma Aldrich), 4 mg/mL collagenase IV (Worthington), and 15 mM HEPES media (Gibco)] and were mechanically dissociated and then resuspended. The sciatic nerve tubes were then incubated on a shaker at 37 °C for 45 min. Following enzymatic digestion, cells were filtered using a 70-µm cell strainer and washed two times with 0.1 M PBS + 2% FBS. The cells were resuspended in PBS with 2% FCS and the protein transport inhibitor cocktail (1:500) and incubated on a shaker for 4 h at 37 °C. At the end of the incubation, the cells were directly fixed with 1.6% PFA for 10 min on a shaker at room temperature, washed two times with 0.1M PBS with 2%FCS, and frozen at –20 °C. For macrophage analysis, three mice were pooled and analyzed in one experiment.

### Mouse CyTOF Experiment.

Antibody testing, panel design and antibody conjugation, and cell staining were performed as described in our previous publication (22). For more details, see **SI Appendix*, Materials and Methods*.

### Human Postmortem Single-Cell Isolation.

Fresh human brain tissues were provided from the donor to the Oregon Brain Bank (Oregon, USA) following the consent of next of kin and deidentification of tissues as stipulated by the IRB to conform to the requirements of nonhuman subject research on human postmortem tissue (*SI Appendix*, Table S5). Immediately following the autopsy, the motor cortex and cervical spinal cord tissues were dissociated using Dounce homogenization in cold 0.1 M HBSS (Gibco) on ice. The homogenized tissue was filtered through a 70-μm cell strainer (Falcon) and centrifuged at 750 g for 7 min at 4 °C. Pelleted cells were then resuspended in 5 mL of 0.1 M HBSS + 2 mL of RPMI (Gibco) + 3 mL of isotonic Percoll [90% Percoll (GE Healthcare) + 10% 1 M HBSS], then a 2-mL layer of 70% Percoll was slowly layered to the bottom of the tube using a 3.5-inch spinal needle (BD Biosciences). Cells were then centrifuged at 500 g for 15 min at RT without break. Single cells were isolated from the interface between the 30% and 70% Percoll layers. Isolated cells were then washed twice with 0.1 M PBS (Gibco) with 2% heat-inactivated fetal bovine serum (FBS) (Atlas Biologicals). The cells were fixed with 0.2 μm-filtered 1.6% paraformaldehyde (electron microscopy), shaken for 10 min at RT, and then washed twice with 0.1 M PBS supplemented with 2% FBS. Fixed cells were stored at −20 °C until use.

### Human Postmortem Tissue CyTOF Analysis.

Frozen isolated microglia were thawed on ice, washed twice at 500 g for 5 min at 4 °C using 0.1 M Maxpar PBS (Fluidigm) supplemented with 2% FBS, refiltered through a 70-μm cell strainer (Falcon), and counted under a microscope. Cells were pelleted down and washed twice with 1 mL Maxpar Cell Staining Buffer (CSB) (Fluidigm) at 500 g for 5 min at 4 °C. The cells were then resuspended in 50 μL CSB, and a surface antibody cocktail (*SI Appendix*, Table S6) created in 50 μL CSB was added. The cells were incubated in antibodies for 45 min at RT on a shaker. After incubation, the cells were washed twice in CSB and spun down at 500 g for 5 min at 4 °C. After staining, the cells rested for 15 min at 4 °C, and 500 μL cold methanol (Fisher Chemical) was added for 20 min at 4 °C. Once permeabilized, the cells were washed twice with CSB, pelleted down at 500 g for 5 min at 4 °C, and resuspended in 100 μL CSB. The cells were then incubated overnight at 4 °C in a solution of 0.1 μM 191/193Ir DNA intercalator (Fluidigm) and 0.2 μm-filtered 1.6% paraformaldehyde diluted in 0.1 M Maxpar PBS. Prior to CyTOF acquisition, the cells were washed once with cell staining media and then two times with double-deionized water (ddH_2_0). EQ Four Element Calibration Beads (Fluidigm) containing known concentrations of 140/142Ce, 151/153Eu, 165Ho, and 175/176Lu were added at 1:10 to each sample. The cells were analyzed on a CyTOF Helios™ (Standard BioTools) at an event rate of 200 to 300 cells per second. All mass cytometry files were normalized together using the mass cytometry data normalization algorithm. Stained cells were analyzed on a Helios mass cytometer (Fluidigm) at an event rate between 50 and 200 cells per second. Sample normalization was performed using normalization beads. Further analysis was performed using Cell Engine (https://cellengine.com).

### Tissue Collection for Histology.

#### α5 integrin antibody– and isotype control antibody–treated SOD1^G93A^ mice.

Mice were anesthetized by intraperitoneal injection of pentobarbital (600 mg/kg) and transcardially perfused with 0.9% saline followed by 4% paraformaldehyde in phosphate buffer (PB) (pH = 7.4). Following perfusion, the spinal cord was removed and immersed in 4% paraformaldehyde for 2 h at room temperature. Samples were then transferred in 15% sucrose in 1X PBS and stored at 4 °C. Tissues were then embedded in OCT medium and frozen in dry ice-cooled isopentane before long-term storage at −80 °C.

#### Untreated SOD1^G93A^ and C57Bl/6J mice.

Mice were anesthetized with an intraperitoneal injection of ketamine (100 mg/kg). The mice were first transcardially perfused with 20 mL 1XPBS, followed by 50ml 4% paraformaldehyde (electron microscopy). Spinal cords and sciatic nerves were dissected out and immersed in 4% paraformaldehyde for postfixation for 4 h. Tissues were then transferred in 30% sucrose in 1X PBS for 48 h before freezing in OCT in dry ice-cooled isopentane. The tissues were stored at −80 °C.

### Histology.

#### Spinal cord from anti-α5 integrin antibody– and isotype control–treated SOD1^G93A^ mice.

(Two groups: n = 6/group): Six cryosections from spinal cord tissue array were cut at 10 μm thickness with a Leica cryotome (CV5030) and placed on a glass slide. The next 14 sections per level were discarded. This collection scheme was repeated for five levels. The sections were stored at −20 °C.

#### Spinal cord and sciatic nerve from untreated SOD1^G93A^ and C57Bl/6J mice.

Thirty micrometers of transverse floating serial sections was obtained using a Leica cryostat (CM1850) and dispatched in a 24 well-plate, each well filled with 500 μL 1XPBS. Sections were stored at 4 °C in 1X PBS, 0.03% azide. Twelve micrometers of longitudinal serial sciatic nerve sections (left or right) was sectioned using a Leica cryostat (CM1850). The sciatic nerve from each mouse was distributed over eight slides in a serial manner.

### Immunofluorescence.

Spinal cord sections were incubated in a blocking solution containing 0.1 M PBS, 0.3% Triton X-100 (PBST), and 3% BSA (Sigma Aldrich) for 1 h at room temperature. Spinal cord transverse cryosections were incubated overnight at room temperature with a rabbit anti-Iba1 (1:500, Wako Chemicals) and a rat anti-α5 integrin (1:400, BioLegend) in PBST. Anti-Iba1 antibody was revealed with a goat anti-rabbit Alexa Fluor-594 secondary antibody (Life Technologies), anti-α5 integrin antibody was revealed with a goat anti-rat Alexa Fluor-488 secondary antibody (Life Technologies) and sections were stained for DAPI before mounting the slides. Sciatic nerves were incubated in a blocking solution containing 0.1 M PBS, 0.3% Triton X-100 (PBST), and 3% BSA (Sigma Aldrich) for 1 h at room temperature. Sciatic nerves were sequentially stained first with a rat anti-α5 integrin antibody (1:400, BioLegend) overnight at room temperature and revealed with a goat anti-rat Alexa Fluor-488 secondary antibody (Life Technologies). On the second day, sciatic nerves were incubated with a cocktail of rat anti-CD11b (1:400, BD Biosciences), rat anti-CD68 (1:400, AbD Serotec), and rat anti-F4/80 (1:100, AbD Serotec) antibodies overnight at room temperature and revealed with a goat anti-rat Alexa Fluor-594 secondary antibody (Life Technologies).

### Image Acquisition and Quantification.

Image of spinal cords and sciatic nerve were obtained using a Zeiss Apotome.2 on an Axio Imager with Zen v3.5 software. All image analyses were performed using Fiji software (ImageJ).

#### Analysis in anti-α5 integrin antibody (MFR5)– and isotype control–treated SOD1^G93A^ mouse spinal cords.

Five slides per mouse, each of them containing one level of lumbar spinal cord, were stained and analyzed for a total of 10 sections per animal. Iba1-positive microglia count and the percentage of α5 integrin– positive microglial cells in the spinal cord were determined by counting the number of cells positive for Iba1 and DAPI only or positive for Iba1, DAPI, and α5 integrin in the ventral horn of the spinal cord sections. Spinal cord microglial Iba1 positivity was measured using a common microglial cell detection threshold for every section to obtain the percentage area (measuring the Iba1^+^ fluorescent immunoreactivity area in the ventral horn parenchyma) in Fiji Software.

#### Analysis in the spinal cord and sciatic nerve from untreated SOD1^G93A^ and C57Bl/6J mice.

For spinal cord, measurements were made from every 24th 30-μm lumbar spinal cord section, which corresponded to a total of 9 to 11 sections per animal. The percentage of α5 integrin–positive microglial cells in the spinal cord was determined by counting the number of cells positive for Iba1 and DAPI only or positive for Iba1, DAPI, and α5 integrin in the ventral horn of the spinal cord sections. For sciatic nerves, measurements were made from every eighth 12-μm sciatic nerve section, which corresponded to a total of four to six sections per animal. Two random pictures of each sciatic nerve were analyzed for a total of 8 to 12 images per mouse. The percentage of α5 integrin–positive macrophages was determined by counting α5 integrin– and CD11b-CD68-F4/80-positive cells over the total number of CD11b-CD68-F4/80-positive macrophages.

### Disease Stage Analysis.

Disease time points were defined as follows. The time of disease onset was retrospectively determined as the time when mice reached peak body weight. The time of early symptomatic stage was defined as the age at which the animals had lost 10% of their maximal weight. Mice were checked daily when they approached end stage, which was defined by paralysis so severe that the animal was unable to right itself within 20 s when placed on its side. Mice of each group were weighed weekly. Kaplan–Meier analysis was used for survival curves. Log-rank tests were used for statistical analysis.

### Pharmacokinetics.

Male C57Bl/6J mice (Jackson Laboratories) were habituated at least for 1 wk prior to study start. Only animals in apparently good health condition were included in the study. All animals were randomly assigned to starting groups (cohort). Injections were initiated at 8 wk of age. Ultra-LEAF™ Purified anti-mouse α5 integrin Antibody Clone: 5H10-27 (MFR5) was purchased from BioLegend, was diluted in PBS (Lonza; 1X w/o Ca++, Mg++) to 1 mg/mL concentration, and administered once by IP route at a volume of 10 μL/g body weight at a dose of 5 mg/kg. Twenty mice were divided into five cohorts of four mice each such that each mouse was only bled once in vivo and once terminally. This number of mice sufficed to cover blood draws that were collected at timepoints over a 2-wk period at 1, 2, 4, 8, 24, 72, 168, 240, and 336 h. For more details, see **SI Appendix*, Materials and Methods*.

### Receptor Occupancy.

C57Bl/6 mice were dosed IP at 5 mg/kg of α5 integrin antibody. Blood was collected at predose, 1, 4, and 24 h, and terminal collections were performed at 3 and 7 d, from three mice per time point. Control blood was collected at the same time points from mice that were injected with vehicle (saline) without α5 integrin antibody. For more details, see *SI Appendix*, *Materials and Methods*.

### Human ALS Cases.

Inclusion criteria were ALS as a both clinical and neuropathologic diagnosis. We screened 635 spinal cord levels from 132 ALS patients with sections stained for hematoxylin and eosin (H&E), a myelin stain [Luxol fast blue–periodic acid Schiff (LFB–PAS)], and immunohistochemistry for ionized calcium-binding adaptor molecule 1 (Iba1, 1:3,000, Wako Chemicals USA), a widely used microglial/macrophage marker. Neuropathologic evidence of active neuronophagia was defined as clusters of macrophages in the ventral horn often with an “empty cell bed” where a degenerating neuron had resided. The cohort assessed in the current study consisted of 96 patients with sporadic ALS (negative for the following genetic mutations: *C9ORF72*, *SOD1*, *TBK1*, *TARDBP*), 29 patients with *C9ORF72* (C9-ALS), 5 patients with *SOD1* mutation (SOD1-ALS), 1 ALS patient with a *TARDBP* mutation, and 1 ALS patient with a *TBK1* mutation. Sporadic ALS and C9ORF72-ALS cases had motor neuron pathology as well as neuronal and glial inclusions with TDP-43 immunohistochemistry, while ALS-SOD1 had neuronal aggregates with immunohistochemistry for SOD-1 or SEDI (38) (*SI Appendix*, Table S3).

### Immunohistochemistry of Human Formalin-Fixed Paraffin-Embedded Tissue.

The human tissue was stained with antibodies and conditions as previously described (39) (*SI Appendix*, Table S7). Briefly, immunohistochemistry was performed on 5 µm-thick sections. Paraffin sections were dewaxed in xylene for 3 × 5 min washes, rehydrated in a graded series of ethanol for 3 × 2 min, and washed in distilled dH_2_0. Normal goat serum in tris-buffered saline with Tween® (TBST) was used to block unspecific antibody binding prior to incubation with the primary antibodies. All stains were processed on the DAKO AutostainerPlus (DAKO) using the DAKO Envision^+^ HRP detection system. Two sets of integrin antibodies were further tested (Absolute Antibody and LSBio) to determine reliability and reproducibility prior to staining the cohort favoring LSBio antibody for formalin-fixed human tissues.

### Digital Image Analyses and Quantification.

Whole-slide images were scanned on an Aperio ScanScope slide scanner (Aperio Technologies) producing high-resolution images. Image quantifications were performed using a digital positive pixel algorithm optimized for each histiocyte-associated stain (Iba1, α5 integrin) expressing densities as number of strong positive pixels per mm^2^ in patients and control cases. All annotations, digital algorithm processing, and data exports were done in a blinded fashion.

### Statistics.

#### Mice.

All manual counts were performed in a blinded manner, and no data were excluded from the analysis for statistical reasons. Beam walking test (Fig. 4 *F* and *G* and *SI Appendix*, Fig. S4*D*): mice falling off the beam were excluded from the assay. Mice were randomly assigned to experimental time points and treatments. Statistics were performed using GraphPad Prism (v9). Graphs were presented in mean ± SEM. Normality of data was tested. Statistical analyses were performed using unpaired *t* test or Mann–Whitney two-tailed tests to compare two groups or Kruskal–Wallis one-way ANOVA to compare more than two groups. Log-rank tests were performed to analyze survival curves.

## Supplementary Material

Appendix 01 (PDF)

## Data Availability

All study data are included in the article and/or *SI Appendix*.

## References

[1] P. Soares , Drug discovery and amyotrophic lateral sclerosis: Emerging challenges and therapeutic opportunities. Ageing Res. Rev. **83**, 101790 (2022). 36402404 10.1016/j.arr.2022.101790

[2] A. M. Clement , Wild-type nonneuronal cells extend survival of SOD1 mutant motor neurons in ALS mice. Science **302**, 113–117 (2003). 14526083 10.1126/science.1086071

[3] D. R. Beers , Wild-type microglia extend survival in PU.1 knockout mice with familial amyotrophic lateral sclerosis. Proc. Natl. Acad. Sci. U.S.A. **103**, 16021–16026 (2006). 17043238 10.1073/pnas.0607423103 PMC1613228

[4] S. Boillée , Onset and progression in inherited ALS determined by motor neurons and microglia. Science **312**, 1389–1392 (2006). 16741123 10.1126/science.1123511

[5] C. S. Lobsiger , Schwann cells expressing dismutase active mutant SOD1 unexpectedly slow disease progression in ALS mice. Proc. Natl. Acad. Sci. U.S.A. **106**, 4465–4470 (2009). 19251638 10.1073/pnas.0813339106 PMC2657393

[6] K. Yamanaka , Astrocytes as determinants of disease progression in inherited ALS. Nat. Neurosci. **11**, 251–253 (2008). 18246065 10.1038/nn2047 PMC3137510

[7] S. H. Kang , Degeneration and impaired regeneration of gray matter oligodendrocytes in amyotrophic lateral sclerosis. Nat. Neurosci. **16**, 571–579 (2013). 23542689 10.1038/nn.3357 PMC3637847

[8] A. Chiot , Modifying macrophages at the periphery has the capacity to change microglial reactivity and to extend ALS survival. Nat. Neurosci. **23**, 1339–1351 (2020). 33077946 10.1038/s41593-020-00718-z

[9] M. E. Alexianu, M. Kozovska, S. H. Appel, Immune reactivity in a mouse model of familial ALS correlates with disease progression. Neurology **57**, 1282–1289 (2001). 11591849 10.1212/wnl.57.7.1282

[10] E. D. Hall, J. A. Oostveen, M. E. Gurney, Relationship of microglial and astrocytic activation to disease onset and progression in a transgenic model of familial ALS. Glia **23**, 249–256 (1998). 9633809 10.1002/(sici)1098-1136(199807)23:3<249::aid-glia7>3.0.co;2-#

[11] I. M. Chiu , A neurodegeneration-specific gene-expression signature of acutely isolated microglia from an amyotrophic lateral sclerosis mouse model. Cell Rep. **4**, 385–401 (2013). 23850290 10.1016/j.celrep.2013.06.018 PMC4272581

[12] J. Brettschneider , Microglial activation and TDP-43 pathology correlate with executive dysfunction in amyotrophic lateral sclerosis. Acta Neuropathol. **27**, 417–428 (2012). 10.1007/s00401-011-0932-x PMC3595560 22210083

[13] J. Brettschneider , Microglial activation correlates with disease progression and upper motor neuron clinical symptoms in amyotrophic lateral sclerosis. PLoS One **7**, 13–15 (2012). 10.1371/journal.pone.0039216 PMC3375234 22720079

[14] A. Chiot, C. S. Lobsiger, S. Boillée, New insights on the disease contribution of neuroinflammation in amyotrophic lateral sclerosis. Curr. Opin. Neurol. **32**, 764–770 (2019). 31306211 10.1097/WCO.0000000000000729

[15] A. Atanasio , C9orf72 ablation causes immune dysregulation characterized by leukocyte expansion, autoantibody production, and glomerulonephropathy in mice. Sci. Rep. **6**, 23204 (2016). 26979938 10.1038/srep23204 PMC4793236

[16] A. Burberry , Loss-of-function mutations in the C9ORF72 mouse ortholog cause fatal autoimmune disease. Sci. Transl. Med. **8** (2016). 10.1126/scitranslmed.aaf6038 PMC5024536 27412785

[17] J. G. O’Rourke , C9orf72 is required for proper macrophage and microglial function in mice. Science **1979**, 1324–1329 (2016). 10.1126/science.aaf1064 PMC5120541 26989253

[18] E. Marchlik , Mice lacking Tbk1 activity exhibit immune cell infiltrates in multiple tissues and increased susceptibility to LPS-induced lethality. J. Leukoc Biol. **88**, 1171–1180 (2010). 20651301 10.1189/jlb.0210071

[19] Y. Ito , RIPK1 mediates axonal degeneration by promoting inflammation and necroptosis in ALS. Science **1979**, 603–608 (2016). 10.1126/science.aaf6803 PMC5444917 27493188

[20] B. Ajami, J. L. Bennett, C. Krieger, W. Tetzlaff, F. M. V. Rossi, Local self-renewal can sustain CNS microglia maintenance and function throughout adult life. Nat. Neurosci. **10**, 1538–1543 (2007). 18026097 10.1038/nn2014

[21] A. Martínez-Muriana , CSF1R blockade slows the progression of amyotrophic lateral sclerosis by reducing microgliosis and invasion of macrophages into peripheral nerves. Sci. Rep. **6**, 1–13 (2016). 27174644 10.1038/srep25663 PMC4865981

[22] B. Ajami , Single-cell mass cytometry reveals distinct populations of brain myeloid cells in mouse neuroinflammation and neurodegeneration models. Nat. Neurosci. **21**, 541–551 (2018). 29507414 10.1038/s41593-018-0100-x PMC8629134

[23] A. M. D’Erchia , Massive transcriptome sequencing of human spinal cord tissues provides new insights into motor neuron degeneration in ALS. Sci. Rep. **7**, 10046 (2017). 28855684 10.1038/s41598-017-10488-7 PMC5577269

[24] H. Keren-Shaul , A Unique Microglia Type Associated with Restricting Development of Alzheimer’s Disease. Cell, **169**, 1276–1290 (2017). 28602351 10.1016/j.cell.2017.05.018

[25] Y. Chen, M. Colonna, Microglia in Alzheimer’s disease at single-cell level. Are there common patterns in humans and mice? J. Exp. Med. **218**, e20202717 (2021). 34292312 10.1084/jem.20202717 PMC8302448

[26] T. A. Yednock , Prevention of experimental autoimmune encephalomyelitis by antibodies against α4β1 integrin. Nature **356**, 63–66 (1992). 1538783 10.1038/356063a0

[27] S. Garofalo , Blocking immune cell infiltration of the central nervous system to tame Neuroinflammation in Amyotrophic lateral sclerosis. Brain Behav. Immun. **105**, 1–14 (2022). 35688338 10.1016/j.bbi.2022.06.004

[28] L. Steinman, Blocking adhesion molecules as therapy for multiple sclerosis: Natalizumab. Nat. Rev. Drug Discovery **4**, 510–518 (2005). 15931259 10.1038/nrd1752

[29] A. Vercelli , Human mesenchymal stem cell transplantation extends survival, improves motor performance and decreases neuroinflammation in mouse model of amyotrophic lateral sclerosis. Neurobiol. Dis. **31**, 395–405 (2008). 18586098 10.1016/j.nbd.2008.05.016

[30] J. Brettschneider , TDP-43 pathology and neuronal loss in amyotrophic lateral sclerosis spinal cord. Acta Neuropathol. **128**, 423–437 (2014). 24916269 10.1007/s00401-014-1299-6 PMC4384652

[31] A. G. Barbeito, P. Mesci, S. Boillée, Motor neuron-immune interactions: The vicious circle of ALS. J. Neural. Transm (Vienna) **117**, 981–1000 (2010). 20552235 10.1007/s00702-010-0429-0 PMC3511247

[32] F. Reichert, R. Levitzky, S. Rotshenker, Interleukin 6 in intact and injured mouse peripheral nerves. Eur. J. Neurosci. **8**, 530–535 (1996). 8963444 10.1111/j.1460-9568.1996.tb01237.x

[33] G. K. Tofaris, P. H. Patterson, K. R. Jessen, R. Mirsky, Denervated Schwann cells attract macrophages by secretion of leukemia inhibitory factor (LIF) and monocyte chemoattractant protein-1 in a process regulated by interleukin-6 and LIF. J. Neurosci. **22**, 6696–6703 (2002). 12151548 10.1523/JNEUROSCI.22-15-06696.2002 PMC6758146

[34] Z. Ye, J. Wei, C. Zhan, J. Hou, Role of transforming growth factor beta in peripheral nerve regeneration: Cellular and molecular mechanisms. Front Neurosci. **16**, 917587 (2022). 35769702 10.3389/fnins.2022.917587 PMC9234557

[35] F. Berriat, C. S. Lobsiger, S. Boillée, The contribution of the peripheral immune system to neurodegeneration. Nat Neurosci. **26**, 942–954 (2023). 37231108 10.1038/s41593-023-01323-6

[36] A. Cugurra , Skull and vertebral bone marrow are myeloid cell reservoirs for the meninges and CNS parenchyma. Science **373**, eabf7844 (2021). 34083447 10.1126/science.abf7844 PMC8863069

[37] O. J. Mezu-Ndubuisi, A. Maheshwari, The role of integrins in inflammation and angiogenesis. Pediatr. Res. **89**, 1619–1626 (2021). 33027803 10.1038/s41390-020-01177-9 PMC8249239

[38] R. Rakhit , An immunological epitope selective for pathological monomer-misfolded SOD1 in ALS. Nat. Med. **13**, 754–759 (2007). 17486090 10.1038/nm1559

[39] K. F. Bieniek , Tau pathology in frontotemporal lobar degeneration with C9ORF72 hexanucleotide repeat expansion. Acta Neuropathol. **125**, 289–302 (2013). 23053135 10.1007/s00401-012-1048-7 PMC3551994

