# Supplementary material for: Elevated α5 integrin expression on myeloid cells in motor areas in amyotrophic lateral sclerosis is a therapeutic target
Source: Proc Natl Acad Sci U S A. 2023 Jul 31;120(32):e2306731120. doi: 10.1073/pnas.2306731120 (PMC10410747; doi:10.1073/pnas.2306731120)
Supplement: Supplementary file 1 — Appendix 01 (PDF) [file pnas.2306731120.sapp.pdf]

PNAS

Supporting Information for

Elevated α5 integrin expression on myeloid cells in motor areas in amyotrophic lateral sclerosis is a therapeutic target in ALS.

Aude Chiot*, Shanu F. Roemer*, Lisa Ryner, Alina Bogachuk, Katie Emberley, Dillon Brownell, Gisselle Jimenez, Michael Leviten, Randall Woltjer, Dennis W. Dickson, Lawrence Steinman†, Bahareh Ajami †

* Equal contribution

† Bahareh Ajami, Lawrence Steinman
Email: ajami@ohsu.edu; steinman@stanford.edu

This PDF file includes:

Supporting Material and Methods
Figures S1 to S4
Tables S1 to S7

Supporting Information

Supplementary Material and Methods

CyTOF experiment

For microglial cells: cells were resuspended in PBS with 0.5% BSA and 0.02% NaN_3, and antibodies against CD16/32 were added at 20 µg/ml for 10 min at room temperature on a shaker to block Fc-receptors. Cells were mixed with a cocktail of metal-conjugated surface marker antibodies (**Supplementary Table 1**), yielding 500-µL final reaction volumes, and stained at room temperature for 30 min on a shaker. Following staining, cells were washed twice with PBS with 0.5% BSA and 0.02% NaN_3. Next, cells were permeabilized with 4 °C methanol for 10 min at 4 °C. Cells were then washed twice in PBS with 0.5% BSA and 0.02% NaN_3 to remove remaining methanol. They were stained with intracellular cytokine antibodies (**Supplementary Table 1**) in 500 µl for 30 min at room temperature on a shaker. Samples were then washed twice in PBS with 0.5% BSA and 0.02% NaN_3. Cells were incubated overnight at 4 °C with 1 mL of 1:4,000 $^{191/193}Ir$ DNA intercalator (DVS Sciences/Fluidigm, Markham, ON) diluted in PBS with 1.6% PFA overnight. The following day, cells were washed once with PBS with 0.5% BSA and 0.02% NaN_3 and then two times with double-deionized water (ddH_20). Cells were analyzed on a CyTOF 2 (Fluidigm, Markham, ON) outfitted with a Super Sampler sample introduction system (Victorian Airship & Scientific Apparatus, Alamo, CA) at an event rate of 200 to 300 cells per second. All mass cytometry files were normalized together using the mass cytometry data normalization algorithm.

For sciatic nerve macrophages: cells were resuspended in Cell Staining media (Fluidigm, 201068). Cells were mixed with a cocktail of metal-conjugated cell surface marker antibodies (**Supplementary Table 2**), yielding 100-µL final reaction volumes, and stained at room temperature for 30 min on a shaker. Following staining, cells were washed twice with cell staining media. Next, cells were permeabilized with the BD

Phosflow perm/wash buffer I (BD Bioscience, 1:10, 557885) and incubated for 20 min at 4°C. Cells were then washed and stained with the cytokine antibody cocktail in the BD Phosflow perm/wash buffer I (**Supplementary Table 2**) in 100 µl for 30 min at 4°C on a shaker. Samples were then washed with Cell staining Media. Cells were incubated overnight at 4 °C with 1 mL of 1:5,000 $^{191/193}$Ir DNA intercalator (DVS Sciences/Fluidigm, Markham, ON) diluted in PBS with 1.6% PFA overnight. The following day, cells were washed once with Cell Staining Media and then two times with double-deionized water (ddH_20). For macrophages (fig. 1f lower panel): stained cells were analyzed on a Helios (Standard BioTools) at an event rate of 200 to 300 cells per second. All mass cytometry files were normalized together using the mass cytometry data normalization algorithm.

Behavioral assessment

Vercelli Score: (According to Vercelli et al., 2008). Animals were placed on the testing table and following behavioral parameters were scored: 4 points for full extension of hind legs away from lateral midline when mouse is suspended by its tail, and mouse can hold this for 2 seconds. When suspended 2-3 times for a few seconds, animals should show movement with both hind legs during suspension at least two times. 3 points: Collapse or partial collapse of leg extension towards lateral midline (weakness) or trembling of hind legs during suspension. 2 points: Toes curl under at least twice during walking of 12 inches (30cm), or any part of foot is dragging along cage bottom/table. 1 point: Rigid paralysis or minimal joint movement, foot not for forward motion. 0 points: Mouse cannot right itself within 15 from either side. Scoring started at 8 weeks (baseline) up to 20 weeks of age. Animals that died in the cage or were euthanized due to reaching the endpoint criteria were not given a score of 0, they were excluded from the analysis.

Beam-Walking Test: Motor coordination and balance of mice were evaluated by measuring their ability to traverse a graded series of narrow beams to reach the home cage. The beams consist of long strips of wood (1 m) with a 20- 13- or 10- mm square cross-section. The beams were placed horizontally, 50 cm above the surface, with one end mounted on a narrow support and the other end attached to the home cage into which the mouse can escape. An angle poise light was positioned above the start of the beam. Three training trials were performed prior to testing with three different starting points on the 20mm square beam: close proximity to the home cage (trial 1), in the center of the beam (trial 2), and at the brightly illuminated end of the beam (trial 3). Once the mice were trained, they receive 2 consecutive testing trials on each of the square (except the 20mm square training beam), in each case progressing from the widest to the narrowest beam. The testing trials were videotaped and afterwards evaluated with the Observer XT 10.5. (Noldus). The latency to traverse each beam and the number of slips off each beam were recorded for each trial. Beam walking test was performed at 18 weeks old.

Clinical Signs Score Evaluation: Clinical symptoms were assessed according to different parameters: general health/ activity; general clinical signs, specific clinical signs related to the SOD1^{G93A} mouse models. Scoring started at 16 weeks old and was assessed on a daily basis. **General health and activity:** 0=Smooth and shiny fur, normal activity; 1=Rough hair coat, cloudy eyes; 5=Dirty hair coat, abnormal posture, reduced activity; 10=Dehydration, hunched posture, isolation. **Clinical findings:** 0=Normal temperature and respiration; 1=Slight deviation of the normal situation; 5=Cold extremities, rapid breathing; 10=Moderate alteration of temperature or breathing; 20=Severe alteration of temperature or respiration. **Line specific:** 0=Physiological movement; 1=Beginning

tremor wobbly walk; 5=Dragging of one or more leg, tremor; 10=Paralysis of one leg; 15=Paralysis of more than one leg; 20=Loss of righting reflex. **Sum of scores:** 0-103.

Pharmacokinetics

In vivo blood (approximately 100 µl per bleeding) was collected by mandibular bleeding from the facial vein/artery plexus without anesthesia at two time points, before treatment start (Predose) and 1 h (± 5 min), 2 h (± 5 min), 4 h (± 5 min) or 8 h (± 5 min), each after treatment. Terminal sampling was performed after 24 h (± 30 min), 72 h (± 1 h), 168 h (± 1 h), 240 h (± 1 h) and 336 h (± 1 h), each after treatment. For terminal sampling animals were anesthetized by IP injection of Pentobarbital (600mg/kg). CSF sampling was performed after confirmation of deep anesthesia and was obtained by dissection of the muscles and exposure of the foramen magnum. Upon exposure, a Pasteur pipette was inserted in an approximate depth of 0.3 - 1 mm into the cisterna magna. CSF was collected by suction and capillary action until flow fully ceased in 0.2 ml polypropylene PCR tubes. For serum collection the thorax was opened, and blood was collected by heart puncture with a 23-gauge needle. Collected blood was then transferred into serum gel clotting activator micro tube (from SARSTEDT). After incubation for at least 20 minutes (60 min. maximum) at RT, serum was prepared from the blood samples by centrifugation (10000 × g, 5 minutes, room temperature). The mouse work was performed at QPS (Austria GmbH). The concentration of anti-α5 integrin antibody was measured using an ELISA whereby serum or CSF was diluted into 1x Tris-buffered saline (TBS; Thermo Scientific J75892-K2) with 1% BSA (Blocker BSA in TBS; Thermo Scientific 37520) and incubated in Immulon 4 HBX (Thermo Scientific 3855) plate wells coated with 0.75 ug/mL recombinant mouse α5β1 integrin protein (R&D 7728-A5) overnight at 4°C and blocked with 2% BSA in 1xTBS. Anti-α5 integrin antibody bound to the mouse integrin was detected using a goat-anti-rat antibody conjugated with biotin

(Novex A18869) at 1:8000 dilution followed by incubation with Streptavidin-polyHRP (Pierce 21140) and then addition of TMB substrate (Thermo Scientific N301). Each step was preceded by three washes with 1x TBS + 0.05% Tween 20. After addition of ELISA Stop Solution (Invitrogen SS04) absorbance at 450nm was measured and concentrations were calculated by interpolation from a standard curve using GraphPad Prism.

Receptor occupancy

After blood collection, 50 ul of blood was plated in duplicate into a 96-well PCR plate. One well to measure Total receptors, and the other well to measure Bound receptors. For measuring Total receptors, a saturating concentration of α5 integrin (1 ug/mL) antibody was added to the wells and incubated at 4°C for 45 minutes. Then 20 µg/mL of anti-rat IgG2a conjugated to Alexa 647 (BioLegend #407512) was added to all wells to detect levels of bound α5 integrin antibody, followed by a panel of antibodies used to distinguish cell types including anti-CD3 Alexa 488 (BioLegend #127645), anti-NK-1.1 BV 421 (BioLegend #108732), CD4 BV 421 (BioLegend #100438); CD11b BUV 395 (BD Biosciences #563553). The samples were incubated at 4°C for 30 minutes. At the end of the incubation period, the red blood cells were lysed, and the cells were fixed using BD Lyse/Fix. Samples were incubated for 10 minutes at room temperature, followed by two washes with PBS + 0.5% BSA. Samples were run on a BD LSR II instrument that was calibrated using BD CS&T beads. The cells were first gated on singlets using FSC-A by FSC-H to exclude any aggregates or doublets. Within this gate, a size gate is drawn to exclude red blood cells and debris. To narrow down the populations of interest, the high SSC-A cells (granulocytes) were excluded in the low/mid SSC gate. The T cells were gated from the previous gate as CD3+/CD4+ for CD4+ T cells and CD3+/CD4- for CD8+ T cells. NK cells were identified as CD3-/NK-1.1+. The negative population remaining

was identified as either T cells or NK cells. Following this gate, Monocytes were identified as mid SSC-A and CD11b+. To calculate receptor occupancy the MFI value from the sample measuring bound receptors was divided by the MFI value from the sample measuring total receptors to derive the metric Bound/Total MFI Ratio. Comparative Biosciences (Sunnyvale, CA) performed the mouse dosing and blood collections. The receptor occupancy analysis was performed at Primity Bio (Fremont, CA).

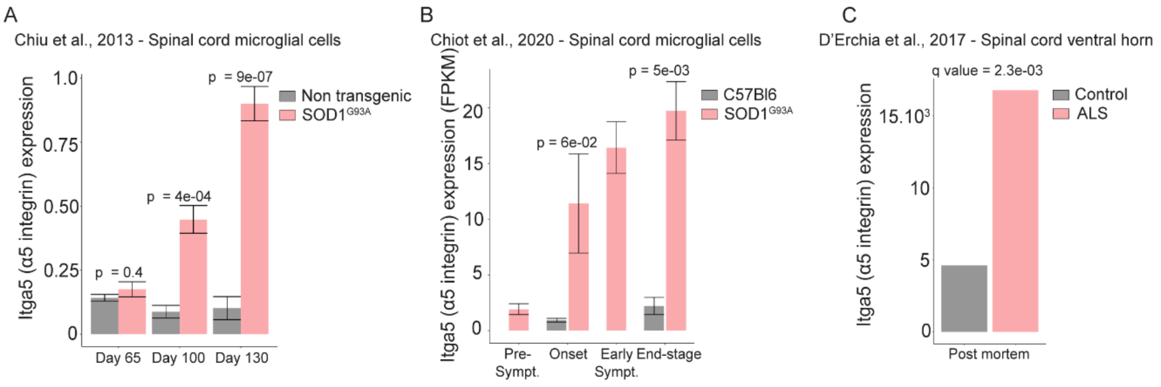

Fig. S1. Expression of α5 integrin is increased during disease in ALS mice and in ALS post-mortem tissues compared to controls.

A, α5 integrin RNA profile in microglial cells isolated from SOD1^{G93A} and non-transgenic (C57BL/6) spinal cord microglial cells extracted from the data of Chiu et al., 2013 (PMID: 23850290). **B**, α5 integrin RNA profile in microglial cells isolated from SOD1^{G93A} and C57BL6 spinal cord microglial cells extracted from the data of Chiot et al., 2020 (PMID: 33077946). **C**, α5 integrin RNA profile in ventral horn spinal cord from ALS patient and control post-mortem tissues extracted from the data of D'herchia et al., 2017 (PMID: 33077946). p-values determined by unpaired t-test with Welch corrections (**A,B**), q-value provided in the original publication (**C**).

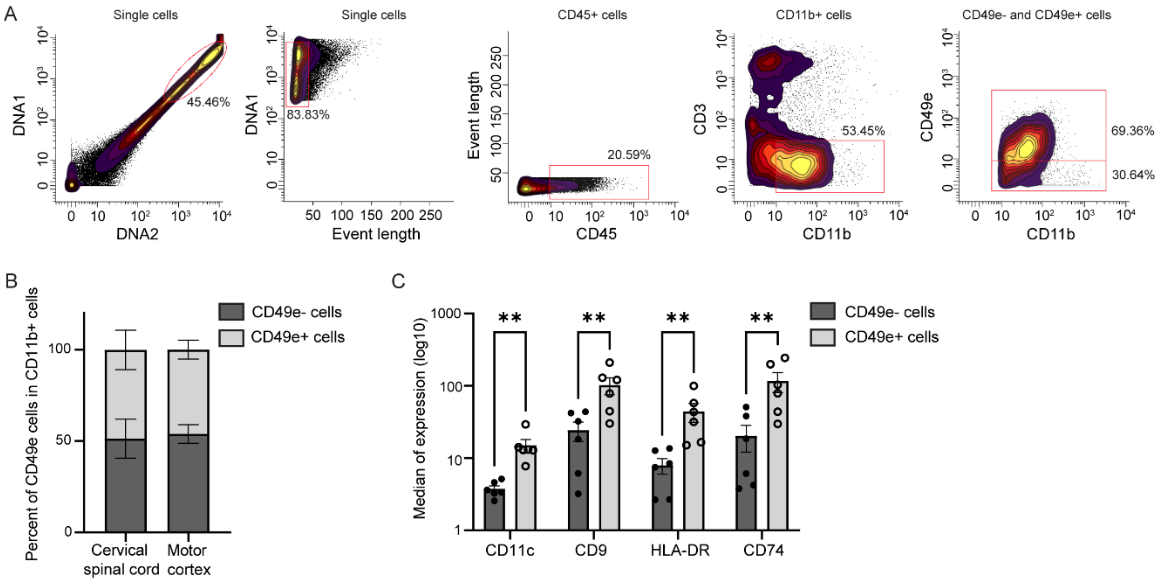

Fig. S2. CyTOF analysis of human myeloid cells expressing α5 integrin.

A) Gating strategy of myeloid cells expressing α5 integrin (CD49e). **B**) Percent of CD11b+ myeloid cells expressing α5 integrin (CD49e) in the cervical spinal cord (n=3) and the motor cortex (n=3) of ALS patients (n=3). **C**) Expressing of CD11c, CD9, HLA-DR and CD74 by α5 integrin (CD49e) – positive and negative myeloid cells in the cervical spinal cord and motor cortex of ALS patients (pooled – n=6). Data are shown as means ± S.E.M., ** P<0.01, determined by multiple Mann-Whitney tests (**C**).

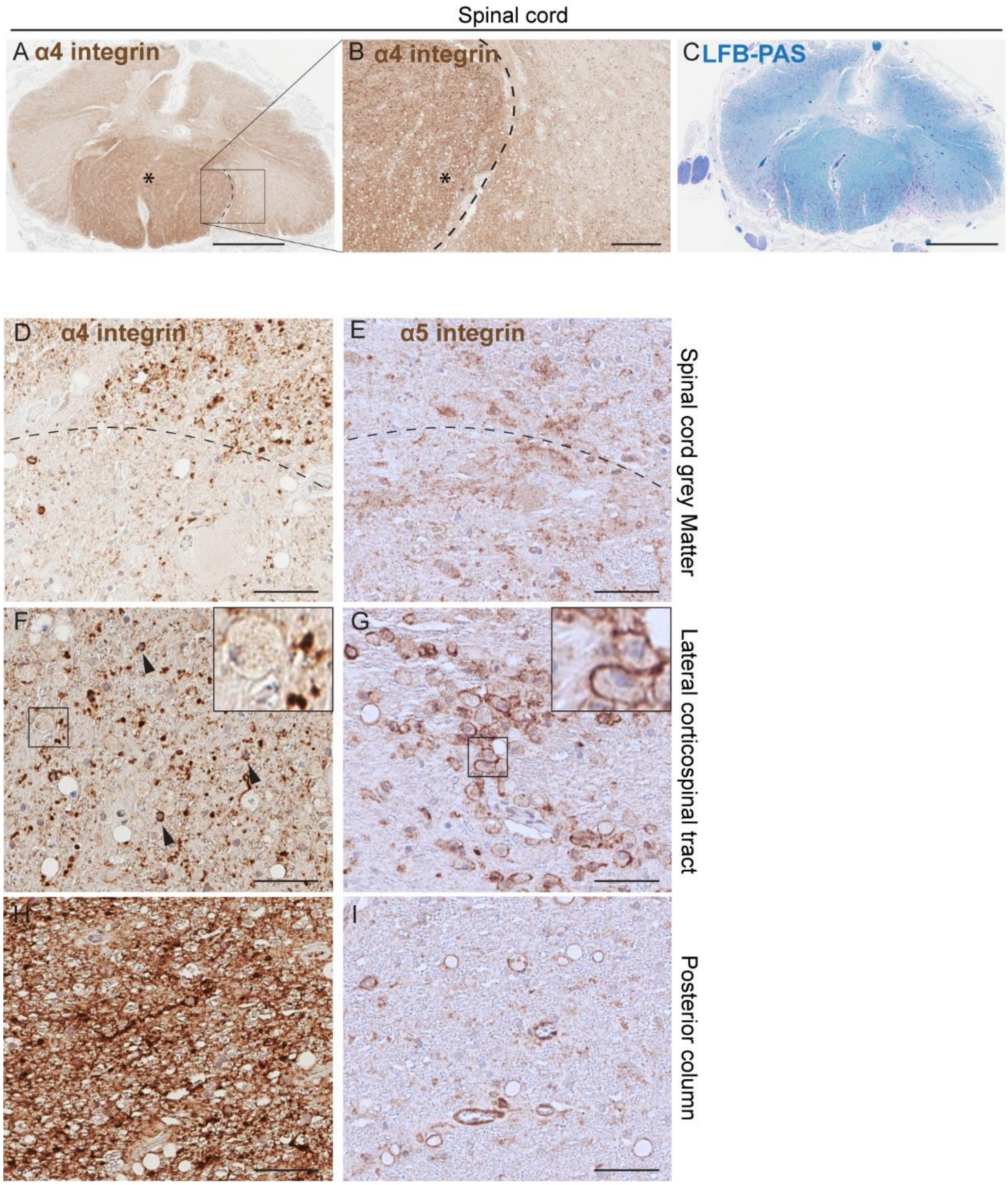
Spinal cord
A α4 integrin
B α4 integrin
C LFB-PAS
D α4 integrin
E α5 integrin
Spinal cord grey Matter
F
G
Lateral corticospinal tract
H
I
Posterior column

Fig. S3. Expression of α5 integrin compared to α4 integrin in ALS.

A-C) A spinal cord whole section (**A**) and boxed area and inset (**B**) shows preserved α4 integrin in the dorsal columns (*) suggesting myelin expression of α4 integrin (**C**). Scale bars: 2 mm (**A,C**), 300 μm (**B**). **D-I)** Comparison of α4 integrin and α5 integrin expression in ALS spinal cord ventral horn, seen below the dashed line (**D,E**), in the lateral corticospinal tract (**F,G**) and in the posterior column (**H,I**) highlighting α4 integrin expression in myelin and oligodendroglia (arrowheads), but not in foamy macrophages (inset), while α5 integrin is specific to foamy microglial cells in motor areas and in the vasculature. Scale bars: 60 μm.

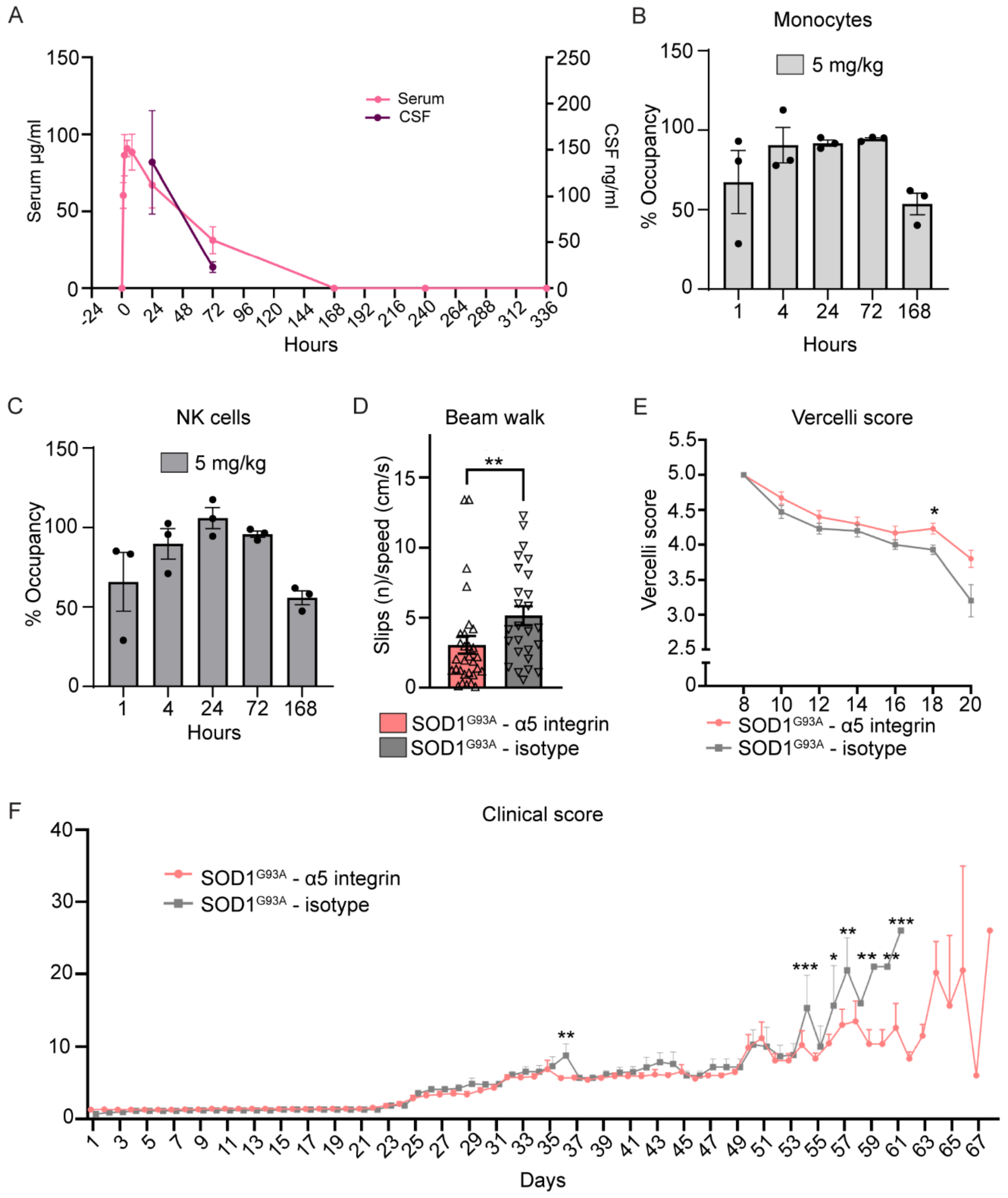
A
Serum
CSF
Serum µg/ml
CSF ng/ml
Hours
B
Monocytes
5 mg/kg
% Occupancy
Hours
C
NK cells
5 mg/kg
% Occupancy
Hours
D
Beam walk
Slips (n)/speed (cm/s)
SOD1G93A - α5 integrin
SOD1G93A - isotype
E
Vercelli score
Vercelli score
SOD1G93A - α5 integrin
SOD1G93A - isotype
F
Clinical score
SOD1G93A - α5 integrin
SOD1G93A - isotype
Days

Fig. S4. Anti-α5 integrin treatment targets peripheral myeloid cells and improve motor phenotype in ALS.

A) Pharmacokinetic study of α5 integrin antibody (MRF5 clone) at the concentration of 5mg/kg in mouse serum (left axis, measured in µg) and mouse CSF (right axis, measured in ng). **B-C)** Receptor occupancy study of α5 integrin antibody (MRF5 clone) at the concentration of 5mg/kg in monocytes (**B**) or Natural killer cells (**C**). **D)** Motor balance assessment by the beam walk test: number of slips per speed (in cm/s) for $SOD1^{G93A}$ mice injected with anti-α5 integrin treatment (pink, n=30) or isotype control (gray, n=26). Mice falling off the beam were excluded: n=4 in isotype control treated group. Data are shown as means ± S.E.M. **E)** Weekly motor symptom assessment using the Vercelli score for $SOD1^{G93A}$ mice injected with anti-α5 integrin treatment (pink) or isotype control (gray) from 8 to 20 weeks old. n=30 per group except at the 20 weeks' time-point: 3 animals in isotype treated group (grey) were euthanized due to reaching disease endpoint, n=27 in isotype control treated group. Score 5: full extension of hind legs, to score 0: complete paralysis without the ability to right itself (for a detailed scoring see methods). Data are shown as means ± S.E.M. **F)** Clinical score assessed daily for $SOD1^{G93A}$ mice injected with anti-α5 integrin treatment (pink) or isotype control (gray) from 112 to 180 days-old Score 0: normal mouse, to score 20: complete paralysis, see material and methods for detailed scoring. n=30 per group until 141 days-old, after, number of mice decreases as they die.

Data are shown as means ± S.E.M., * $P<0.05$, ** $P<0.01$, *** $P<0.001$ Mann Whitney test (**D**), two-way ANOVA and Bonferroni´s multiple comparisons test (**E**), mixed-effect analysis and Bonferroni´s multiple comparisons test (**F**).

Table S1. CyTOF panel from Ajami et al., 2018 for Figs. 1A and 1F

Cell surface

Protein	Clone	Manufacturer	Metal isotope	Concentration (µg/ml)	Catalog number
B220	RA3-6B2	BioLegend	Pr141	2	103202
CD11b	M1/70	BioLegend	Nd142	0.5	101202
CD11c	N418	BioLegend	Nd143	8	117302
CD194	2G12	BioLegend	Gd160	8	131202
CD195	HM-CCR5(7A4)	eBioscience	Gd155	8	14-1951-85
CD200R	OX2R	BioLegend	Yb172	8	123902
CD206	MR5D3	AbD Serotec	Er166	8	MCA2235
CD217	PAJ-17R	eBioscience	Lu175	4	12-7182-82
CD274	B7-H1	BioLegend	Nd146	2	124302
CD3	145-2C11	BioLegend	In113	4	100302
CD38	90	BioLegend	Dy161	4	102702
CD39	Duha59	BioLegend	Er170	3	143802
CD4	RM4-5	BioLegend	Nd150	1	100506
CD45	30-F11	BioLegend	Yb176	1	103102
CD49d	9C10 (MRF4.B)	BioLegend	Sm147	4	103708
CD49e	5H10-27 (MRF5)	BioLegend	Nd148	4	103801
CD80	16-101	BD Pharmingen	Er168	4	553766
CD86	GL-1	BioLegend	Tb159	4	105002
H-2	M1/42	BioLegend	Nd145	1	125502
Ly6c	HK1.4	Novus Biologicals	Eu151	1	NBP1-28046
Ly6g	1A8	BioLegend	Ce140	2	127632

MHCII	M5/114.15.2	BioLegend	In115	2	107602
CD317	120GB	Novus/imgenex	Eu153	4	DDX0390-067
TIM4	Kat5-18	Hycult biotech	Dy163	8	11550M0512
MerTK	Polyclonal	R&D	Dy162	4	DGS0213111
AXL	Polyclonal	R&D	Er167	4	CTC0213041
Trem2	78.18	BioRad	Tm169	4	1113
4D4		Gift from Dr Butovsky	Sm154	0.5	Gift
Fcrls		Gift from Dr Butovsky	Sm152	2	Gift
GFP	FM264G	BioLegend	La139	4	338002

Cytokines

IFN- α	F1	Hycult biotech	Yb173	4	HM1001
IL-10	JES5-16E3	DVS	Gd158	4	3158002B
GM-CSF	MP1-22E9	BioLegend	Dy164	4	505402
IL-17A	TC11-18H10.1	DVS	Tm169	4	3169005B
IL-6	MP5-20F3	DVS	Er167	4	3167003B
TGF- β	19D8	BioLegend	Yb171	4	521704
TNF- α	MP6-XT22	DVS	Dy162	2	3162002B

Table S2. CyTOF panel for Fig. 1G.

Cell Surface

Protein	Clone	Manufacturer	Metal isotope	Concentration (µg/ml)	Catalog number
CD11b	M1/70	BioLegend	Nd142	0.5	101202
CD11c	N418	BioLegend	Nd143	8	117302
CD195	HM-CCR5(7A4)	eBioscience	Gd155	8	14-1951-85
CD200R	OX2R	BioLegend	Yb172	8	123902
CD3	145-2C11	BioLegend	In113	4	100302
CD4	RM4-5	BioLegend	Nd150	1	100506
CD45	30-F11	BioLegend	Yb176	1	103102
CD49e	5H10-27 (MRF5)	BioLegend	Nd148	4	103801
Ly6c	HK1.4	Novus Biologicals	Eu151	1	NBP1-28046
Ly6g	1A8	BioLegend	Ce140	2	127632
MHCII	M5/114.15.2	BioLegend	In115	2	107602
4D4		Gift from Dr Butovsky	Sm154	0.5	Gift

Cytokines

IFN- α	F1	Hycult Biotech	Yb173	4	HM1001
IL-10	JES5-16E3	DVS Sciences	Gd158	4	3158002B
GM-CSF	MP1-22E9	BioLegend	Dy164	4	505402
IL-6	MP5-20F3	DVS Sciences	Er167	4	3167003B
TGF- β	19D8	BioLegend	Yb171	4	521704
TNF- α	MP6-XT22	DVS Sciences	Dy162	2	3162002B

Table S3. ALS Demographics.

Demographics	ALS cohort (N=18)					
	SOD1 (N=5)	C9ORF72 (N=5)	TARDBP (N=1)	TBK1 (N=1)	Sporadic (N=6)	ALS combined
F:M	1.5:1	1:1.5	0:1	1:0	1:2	1:1
Race	White	White	White	White	White	White
Age (median in years)	42-66 (55)	49-77 (66)	59 (NA)	72 (NA)	48-82 (66)	42-82 (61)
Duration (median) years	1-5 (2)	1-4 (1.5)	1	2	1-4 (2)	1-5 (2)
Familial history ALS	5/0	3/2	0/1	0/1	1/5	8/10
**Treatment**						
Riluzole	3	2	0	0	3	8
Edaravone	2	0	0	0	2	4
Mesenchymal Stem cell therapy	0	0	0	0	1	1
Antisense oligonucleotide	1	1	0	0	0	2
Acetyl L carnitine 1	1	1	0	0	0	2
prednisone	1	1	0	0	0	2
No therapy	1	1	1	1	1	5

F: Female, M: Male; N/A = Not applicable

Table S4. Control demographics

Demographics	Normal (N=10)	Other neurologic disease “Non-MND” (N=8)	Combined controls
F:M	1:1	3:1	1:1
Race	Caucasian	Caucasian	Caucasian
Age (median) years	28-79 (64.5)	59-99 (68)	28-99 (66.5)
Duration (median) years	NA	6.5	6.5
Spinal cord	10	0	10
Motor cortex	0	8	8
Familial history ALS	0	0	0

Other neurologic disease: Stroke (n=2), Alzheimer’s disease (n=2), Progressive supranuclear palsy (n=4, with Primary Lateral Sclerosis n=2). Normal: Cognitively normal without motor neuron disease of other neuropathological findings (n=10). F:M; F = Female, M = Male; Y/N = Yes/No; NA = Not applicable

Table S5. ALS demographics for CyTOF analysis

Demographics	ALS cohort (N=3)	
	C9ORF72 (N=1)	Sporadic (N=2)
F:M	1:0	1:1
Race	White	White
Age	54	75-77
Duration years	10	0.5-1.5
Familial history ALS	1	0/2

Table S6. Antibodies used for Human *post-mortem* CyTOF analysis

Protein	Clone	Manufacturer	Metal isotope	Concentration (µg/ml)	Catalog number
CD45	HI30	Fluidigm	Y89	0.3	3089003B
CD11b	ICRF44	Fluidigm	Bi209	0.3	3209003B
CD3	UCHT1	Fluidigm	Pr141	0.3	3141019B
CD81	5A6	Fluidigm	Nd145	0.3	3145007B
CD8a	RPAT8	Biolegend	Nd146	0.3	301002
CD44	IM7	Fluidigm	Nd150	0.3	3150018B
CD14	M5E2	Fluidigm	Eu151	0.3	3151009B
CD95	DX2	Fluidigm	Sm152	0.3	3152017B
Galectin-3	M3/38	Fluidigm	Eu153	0.3	3153026B
CD163	GHI/61	BioLegend	Sm154	0.3	3154007B
CD11c	3.9	BioLegend	Gd157	0.3	301616
CD33	WM53	Fluidigm	Gd158	0.3	3158001B
CD9	SN4 C3-3A2	Fluidigm	Yb171	0.3	3171009B
CD74	LN2	Fluidigm	Er166	0.3	3166018B
CD49e	NKI-SAM-1	Fluidigm	Gd160	0.3	3160015B
CD354	TREM-26	Fluidigm	Yb172	0.3	3172022B
CD172a	SE5A5	Fluidigm	Lu175	0.3	3175024B
HLA-DR	L243	Fluidigm	Yb173	0.3	3173005B
TFN-a	Mab11	Fluigigm	Cd114	0.3	3114002C
IL-6	MQ2-13A5	Fluidigm	Cd106	0.3	3106003C
IL-10	JES3-9D7	Fkuidigm	Ho165	0.3	3165044C
GM-CSF	BVD2-21C11	Fluidigm	Tb159	0.3	3159008B

Table S7. Antibodies used for Human *post-mortem* tissue staining

Antibody	Source	Clone	Host/ clonality	Antigen Retrieval	Concentration
ITGAM (CD11b)	LS Bio	LS-C344008	Rabbit/Polyclonal	EDTA, pH 9	1:5000
alpha 4 integrin (CD49d)	LS Bio	LS-A9385	Rabbit/Polyclonal	Citrate, pH 6	1:500
alpha 5 integrin (CD49e)	LS Bio	LS-B4121	Mouse/Monoclonal	Citrate, pH 6	1:2000
IBA1	Wako	019-19741	Rabbit/Polyclonal	None	1:3000
MBP	Chemicon	MAB381	Mouse/Monoclonal	None	1:500
Neurofilament phosphorylated	Covance	SMI31	Mouse/Monoclonal	None	1:40000
Choline acetyl-transferase	Chemicon	AB144P	NA	None	1:500
Phospho-TDP-43	Cosmo	pS409/410	Rabbit/Polyclonal	None	1:5000
